# Deep Sequencing Reveals Novel MicroRNAs and Regulation of MicroRNA Expression during Cell Senescence

**DOI:** 10.1371/journal.pone.0020509

**Published:** 2011-05-26

**Authors:** Joseph M. Dhahbi, Hani Atamna, Dario Boffelli, Wendy Magis, Stephen R. Spindler, David I. K. Martin

**Affiliations:** 1 Center for Genetics, Children's Hospital Oakland Research Institute, Oakland, California, United States of America; 2 Department of Basic Sciences, Neuroscience, The Commonwealth Medical College, Scranton, Pennsylvania, United States of America; 3 Department of Biochemistry, University of California Riverside, Riverside, California, United States of America; New Mexico State University, United States of America

## Abstract

In cell senescence, cultured cells cease proliferating and acquire aberrant gene expression patterns. MicroRNAs (miRNAs) modulate gene expression through translational repression or mRNA degradation and have been implicated in senescence. We used deep sequencing to carry out a comprehensive survey of miRNA expression and involvement in cell senescence. Informatic analysis of small RNA sequence datasets from young and senescent IMR90 human fibroblasts identifies many miRNAs that are regulated (either up or down) with cell senescence. Comparison with mRNA expression profiles reveals potential mRNA targets of these senescence-regulated miRNAs. The target mRNAs are enriched for genes involved in biological processes associated with cell senescence. This result greatly extends existing information on the role of miRNAs in cell senescence and is consistent with miRNAs having a causal role in the process.

## Introduction

The accumulation of senescent cells, and their prolonged activity, disturbs the microenvironment of aging tissues, thereby compromising tissue function and contributing to age-related pathologies [1]–[3]. Hence there is great interest in determining the mechanisms by which senescence contributes to the aging process. Cell senescence, usually accepted as a manifestation of aging at the cellular level, occurs when cultured cells cease to proliferate, remaining viable and metabolically active but undergoing profound changes in gene expression and morphology [1], [4], [5]. Various triggers such as telomere uncapping, DNA damage, and oncogene activation can evoke senescence; such triggers can engage several mechanisms ranging from cell cycle arrest to activation of tumor suppressors. However, all senescing cells undergo profound changes in gene expression [6], [7]. Comprehensive gene expression profiling has identified genes in cell cycle, insulin growth factor, interferon, MAP kinase and oxidative stress pathways as consistently dysregulated during cell senescence [8]. Altered gene expression gives rise to the senescent phenotype, and is well established as part of the mechanisms and pathways that activate the senescence program in cells [7]. However, the factors responsible for the alterations of gene expression during senescence remain elusive.

MicroRNAs (miRNAs) are key modulators of gene expression in various biological and pathological processes [9], [10]. These RNAs, ∼22 nucleotides in size, act as sequence guides that direct Argonaute protein complexes to mRNAs, where they decrease protein synthesis through translational repression or mRNA degradation [11], [12] and thereby influence many basic cellular processes [13], [14] and diseases [15,–17]. Changes in miRNA expression levels occur in cellular senescence and organismal aging [18], [19], and have been linked to changes in levels of mRNAs that are putative targets of specific miRNAs [20]–[22].

To date, studies exploring the role of miRNAs in senescence have relied on microarrays to assay miRNA expression. Deep sequencing, a set of technologies that produce very large amounts of sequence data from nucleic acid specimens, is rapidly replacing microarrays as the technology of choice for quantifying and annotating miRNAs [23], [24]. Deep sequencing has superior ability to capture the scale and complexity of whole transcriptomes [25]. In particular, short read deep sequencing (e.g. Illumina, Solid) is appropriate for miRNAs because a complete miRNA can be sequenced with a single read. While array design relies on knowledge of the miRNAs being interrogated, deep sequencing allows discovery of novel miRNAs. Furthermore, microarray methods lack the dynamic range to detect and quantify low abundance transcripts, but deep sequencing can identify miRNAs that are expressed at levels below the threshold of detection by microarrays. In addition, deep sequencing eliminates background problems that result from cross-hybridization in microarrays, thus facilitating interpretation of the signal and obviating the non-linear data manipulation steps required by microarrays. Therefore, the application of deep sequencing to miRNA studies has the potential to discover novel miRNAs and to detect expression of rare but functionally significant miRNAs. Deep sequencing has not previously been applied to analysis of miRNA expression in cell senescence. In this study, we conducted a deep characterization of miRNA expression in young and senescent IMR90 human fibroblasts, and predicted biological processes possibly regulated by changes in miRNA expression during senescence.

## Results

### Deep sequencing of miRNAs from young and senescent fibroblasts and analysis with miRDeep2

**Figure 1:**
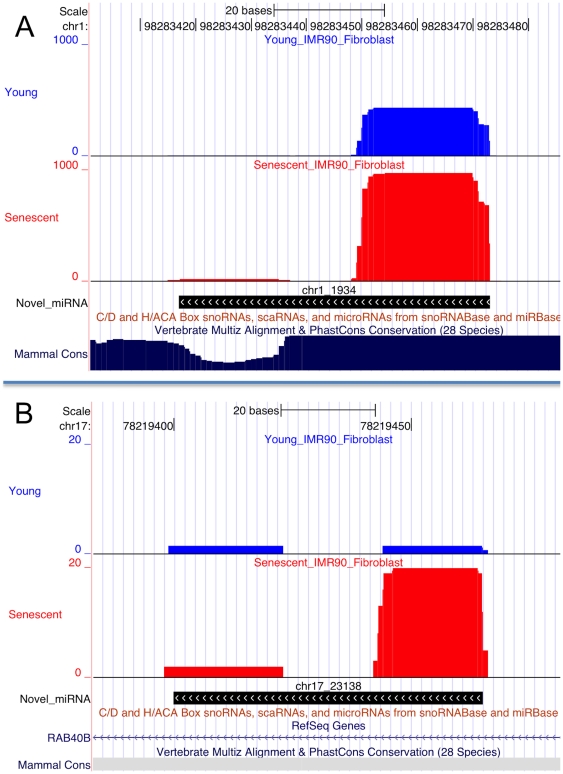
Examples of novel miRNAs discovered by deep sequencing of IMR90 fibroblasts. Expression of both novel miRNAs is increased by senescence. A) Novel miRNA located in a conserved genomic region. Shown are screenshots from the UCSC genome browser, displaying the Illumina sequencing reads (blue: young IMR90; red: senescent IMR 90), and the novel precursor miRNA (black) predicted by miRDeep2 with provisional id chr1_1934 (see Table 1). The mammalian conservation track is at the bottom (deep blue). UCSC genome browser tracks for non-coding RNAs are shown, with no RNAs annotated in this region. The “stacks” of sequence reads identify the mature miRNA, which is more abundant in the senescent IMR90 cells. The coverage depth (number of reads, y-axis) shows few reads mapping to the star region of the miRNA precursor. B) Novel miRNA with provisional id chr17_23138 (see Table 1) predicted to map to an intron by miRDeep2. The same UCSC genome browser tracks as in A are shown. The precursor miRNA is in black. Note the scale on the y-axis: this novel miRNA is less abundant than the example in A.

To investigate miRNA expression during cell senescence, we used Illumina sequencing to generate and analyze 11,382,713 and 9,082,893 raw sequencing reads of small RNAs isolated from young and senescent IMR90 human lung fibroblasts, respectively. The reads were mapped to the human genome (NCBI36/hg18) and analyzed by miRDeep2, an algorithm based on the miRNA biogenesis model. It aligns sequencing reads to potential hairpin structures in a manner consistent with Dicer processing, and assigns log-odds scores to measure the probability that hairpins are true miRNA precursors. The output of this analysis is a scored list of known and novel miRNAs with their expression levels. MiRDeep2 detected 452 known miRNAs that passed the relatively stringent score cut-off of 4, which provides a signal-to-noise ratio of 15.6 (Table S1). (To detect miRNAs in deep sequencing data by miRDeep2, a score cutoff corresponding to a prediction signal-to-noise ratio >10 is often used.) MiRDeep2 predicted 46 potential novel miRNAs at the same score cut-off of 4 (Table S1). Removal of loci matching other RNA genes or genomic repeats reduced this list to 20 candidate novel miRNAs (Table 1). Illustrative examples of novel miRNAs are depicted in Fig. 1. The novel miRNA in Fig. 1A maps to a conserved genomic region, and is not annotated in the ‘Non-coding RNA Genes’ and ‘sno/miRNA’ UCSC tracks. The novel miRNA in Fig. 1B is not conserved, and is derived from an intron of the RAB40B gene. In both novel miRNAs, many more sequencing reads map to the senescent than to the young sample, indicating an induction by senescence.

**Table 1:** Novel miRNAs predicted by miRDeep2 in IMR90 fibroblast data sets.

Novel miRNA Id^1^	Genomic location^2^	MiRDeep2 score^3^	Probability^4^	Total^5^	Mature^6^	Star^7^
chr1_1934	chr1:98283418-98283473:-	768.3	72 +/− 10%%	1505	1485	20
chr12_17303	chr12:107915210-107915253:+	307.8	72 +/− 10%%	602	601	1
chr6_9853	chr6:1335558-1335615:-	47.2	72 +/− 10%%	98	87	11
chr7_11235	chr7:101898924-101898982:+	39.3	72 +/− 10%%	75	74	1
chr11_16723	chr11:121532114-121532177:-	26	72 +/− 10%%	50	30	20
chr2_3531	chr2:208327785-208327842:+	21.1	72 +/− 10%%	40	39	1
chr2_3059	chr2:111795064-111795130:-	17.6	72 +/− 10%%	42	41	1
chr19_24258	chr19:2713642-2713692:-	15	72 +/− 10%%	35	34	1
chr2_4756	chr2:233123445-233123507:-	14.2	72 +/− 10%%	18	17	1
chr12_16965	chr12:47506359-47506417:+	13.8	72 +/− 10%%	33	32	1
chr17_23138	chr17:78219400-78219465:-	12.4	72 +/− 10%%	22	19	3
chr17_21914	chr17:26926413-26926479:+	10.4	72 +/− 10%%	26	25	1
chr2_4865	chr2:239938364-239938425:-	10	72 +/− 10%%	10	9	1
chr5_8282	chr5:9106935-9106998:-	8	74 +/− 9%%	13	9	4
chr15_20416	chr15:72490756-72490821:-	7.4	75 +/− 9%%	13	11	2
chr13_18468	chr13:49450842-49450905:-	6.2	76 +/− 8%%	11	10	1
chr11_16105	chr11:13441281-13441335:-	5.9	75 +/− 8%%	48	48	0
chr6_9790	chr6:150157484-150157534:+	4.9	66 +/− 8%%	48	48	0
chr10_14949	chr10:72362347-72362419:-	4.7	66 +/− 8%%	38	38	0
chr6_9666	chr6:114132759-114132810:+	4.4	66 +/− 8%%	42	42	0

1 A unique identification containing the chromosome and an arbitrary number.

2 Location of the miRNA precursor in the human genome NCBI36/hg18.

3 The miRDeep2 score represents the log-odds probability of a sequence being genuine miRNA precursor versus the probability that it is a background hairpin, given the evidence from the data.

4 The estimated probability that a predicted novel miRNA with a score of this or higher is a true positive.

5 The sum of read counts that map to the predicted mature, loop and star miRNAs. The number of reads that map to the predicted miRNA loop is zero for all listed novel miRNAs.

6 The number of reads that map to the predicted mature miRNA.

7 The number of reads that map to the predicted star miRNA.

### Differentially regulated known miRNAs in young and senescent fibroblasts

**Figure 2:**
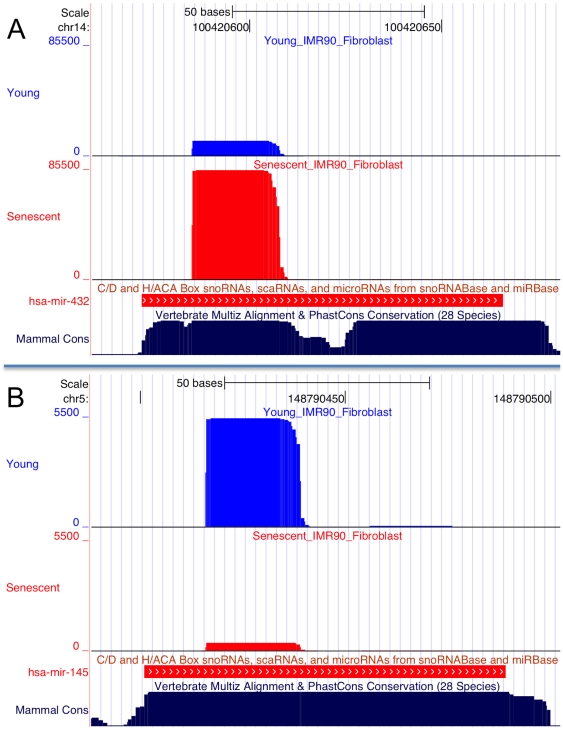
Examples of known miRNAs that are regulated in IMR90 senescence, showing sequence depth. UCSC genome browser views of two miRNAs aligned to the human genome. Reads from young IMR90 cells are shown in blue, and reads from senescent cells in red (number of reads is shown on the y-axis). (A) mir-432; (B) mir-145. The miRNAs (in red) labeled on the snoRNABase/miRBase track represent the miRNA precursors; note that the sequence depth of the mature miRNA, but not the star region, changes in both cases.

**Figure 3:**
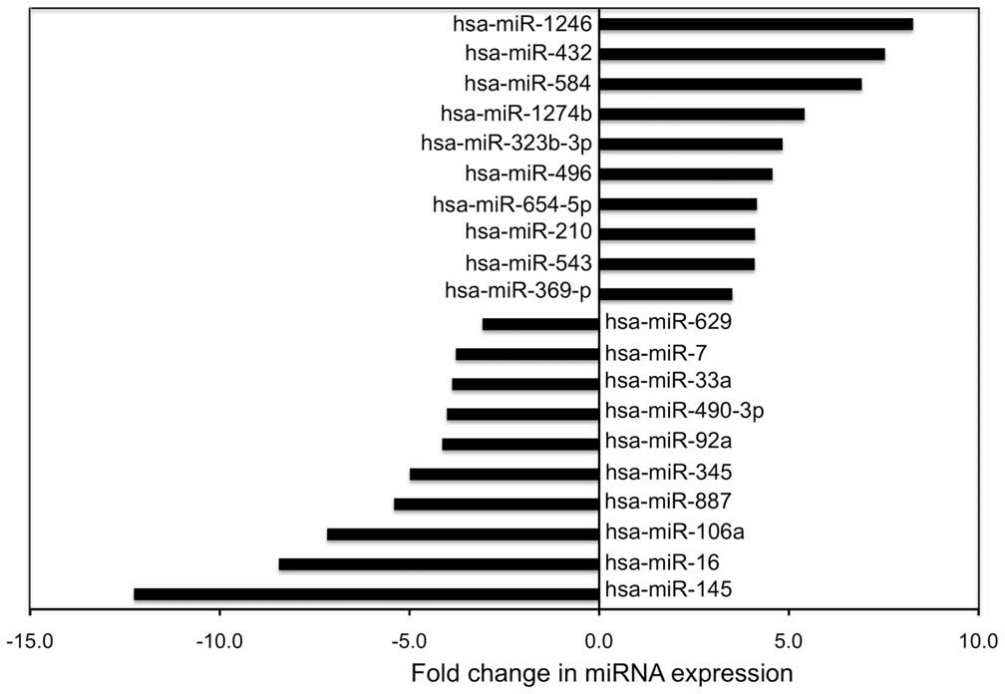
miRNAs differentially regulated in IMR90 senescence. MiRNAs are labeled with miRBase terminology. The x-axis denotes the fold change in sequence reads between young and senescent IMR90 cells: miRNAs overexpressed during senescence have positive values and miRNAs underexpressed during senescence have negative values. These miRNAs were selected for display because they have high expression level (sequence read counts) in at least one of the states (young or senescent) and show a high fold change. For a complete list of differentially expressed miRNAs, see Table S2.

To test for differential miRNA expression between young and senescent fibroblasts, we used expression values generated by miRDeep2 as input for the Bioconductor DESeq package [26]. DESeq uses a negative binomial distribution model to test for differential expression in deep sequencing datasets. 141 miRNAs were induced by senescence and 131 were repressed (Table S2). Many of these have previously been identified as senescence-regulated in microarray studies [20], [27], [28], with some differences that may reflect differences in cell types and senescence models. However the results include many miRNAs not previously identified as senescence-associated: for example, mir-432 is highly induced by senescence (Fig. 2A), mir-145 is repressed (Fig. 2B), and others are depicted in Fig. 3.

### Potential target genes regulated by senescence-induced miRNA expression changes

An individual miRNA may regulate hundreds of mRNAs; this ability to modulate gene expression gives miRNAs considerable influence on physiology and pathology. We computationally identified mRNA targets of miRNAs using the prediction algorithm TargetScan in ExprTargetDB [29], and identified mRNA expression patterns correlated with miRNA expression changes (see materials and methods for details of the steps used in matching miRNA and mRNA expression data). We used affy and limma Bioconductor packages to generate senescence-associated gene expression profiles from Affymetrix and two-color microarray raw data files of young and senescent IMR90 and MRC5 fibroblasts obtained from the GEO repositories GSE19018 and GSE15919, respectively. The differentially expressed genes were separated into upregulated and downregulated genes. We first matched the *in silico* predicted miRNA target genes to the mRNA expression obtained from the Affymetrix microarray study of IMR90 fibroblasts. These comparisons revealed 386 genes potentially downregulated by senescence-induced miRNA overexpression (Table S3), and 131 genes potentially upregulated by senescence-induced miRNA underexpression (Table S4). We then used a three-way Venn diagram to match the *in silico* predicted miRNA target genes to the mRNA expression obtained from the Affymetrix microarray study of IMR90 fibroblasts and the mRNA expression obtained from the two-color microarray study of MRC5 fibroblasts. This comparison revealed 143 genes potentially downregulated by senescence-induced miRNA overexpression in both types of fibroblasts (Table S5), and 36 genes potentially upregulated by senescence-induced miRNA underexpression (Table S6).

### Biological processes regulated by miRNA changes during senescence

To functionally annotate the genes identified as potentially regulated by miRNAs during senescence, we used DAVID and Gene Ontology. DAVID Functional Annotation Clustering feature identifies GO terms associated with genes, and then clusters the most relevant GO terms into smaller and biologically meaningful groups. This functional annotation reveals the biological processes targeted by miRNA expression changes during senescence. Genes repressed by senescence-upregulated miRNAs produced five clusters that have enrichment scores higher than 1.3 (Table 2). Terms related to positive regulation of cell proliferation are by far the most highly represented and enriched. Other enriched biological processes suppressed by miRNA upregulation during senescence include regulation of cellular metabolic processes, nitrogen compound metabolic processes, lung development, and cell differentiation. The KEGG pathway ‘actin cytoskeleton’ was also significantly enriched (Figure S1), suggesting that miRNAs regulate cytoskeletal structure changes that give rise to the enlarged and flattened cell morphology characteristic of the senescence phenotype. Targets of miRNAs downregulated by senescence were also organized into five significant annotation clusters (Table 3), including positive regulation of cell adhesion, negative regulation of apoptosis, and cell cycle arrest. Inhibition of proliferation and resistance to apoptosis are hallmarks of senescence [5], [30], and inhibition of biosynthetic pathways may influence the cessation of growth during senescence. Conversely, stimulation of protein metabolism and modification are consistent with evidence that anabolic processes are enhanced during cellular senescence [31]. Functional annotation of genes identified as potentially regulated by miRNAs during senescence in two types of fibroblasts, IMR90 and MRC5, revealed clusters very similar to the ones obtained from the annotation of genes identified in IMR90 fibroblasts only (Table 4). Notably, senescence-induced changes in miRNA levels seem to be associated with suppression of cell proliferation and cellular metabolic processes and stimulation of cell adhesion in two types of fibroblasts, IMR90 and MRC5.

**Table 2:** Functional annotation clusters of enriched GO biological processes predicted to be suppressed by miRNA upregulation in senescent IMR90 fibroblasts.

Cluster #	GO biological processes	Count^2^	P-Value^3^	MiRNAs^4^
**Cluster 1** (ES = 3.5)^1^	GO:0042127∼regulation of cell proliferation	36	0.0001	hsa-miR-1; 15b; 16; 25; 29a; 30; 30b; 30c; 34a; 103; 128a; 137; 140; 141; 206; 210; 302a; 302b; 302c; 302d
	GO:0008284∼positive regulation of cell proliferation	23	0.0002	
**Cluster 2** (ES = 3.4)	GO:0060541∼respiratory system development	11	0.0002	hsa-miR-10a; 10b; 21; 145; 148a; 148b; 152; 155; 181a; 181c; 190; 196a; 196b; 198; 367; 516b; 524-5p; 576-3p; 590-5p; 630
	GO:0030324∼lung development	10	0.0004	
	GO:0030323∼respiratory tube development	10	0.0005	
**Cluster 3** (ES = 2.8)	GO:0031323∼regulation of cellular metabolic process	108	0.0002	hsa-miR-16; 22; 25; 30; 30b; 30c; 34a; 101; 137; 140; 141; 155; 183; 210; 218; 223; 373; K12-11
	GO:0051171∼regulation of nitrogen compound metabolic process	90	0.0005	
	GO:0031326∼regulation of cellular biosynthetic process	90	0.0008	
**Cluster 4** (ES = 2.6)	GO:0051173∼positive regulation of nitrogen metabolic process	29	0.0008	hsa-miR-1; 7; 10a; 15b; 16; 19a; 25; 30; 34a; 103; 107; 128a; 137; 140; 141; 148a; 148b; 195; 206; 210
	GO:0031328∼positive regulation of cellular biosynthetic process	30	0.0010	
	GO:0031325∼positive regulation of cellular metabolic process	35	0.0017	
	GO:0010557∼positive regulation of macromolecule biosynthetic process	28	0.0020	
**Cluster 5** (ES = 2.1)	GO:0045597∼positive regulation of cell differentiation	12	0.0016	hsa-miR-1; 7; 10a; 19a; 22; 30d; 128a; 134; 140; 148a; 148b; 152; 155; 190; 196a; 196b; 206; 218; 223; 373
	GO:0045595∼regulation of cell differentiation	23	0.0020	

1,2,3,4 are the same as in Table 3.

**Table 3:** Functional annotation clusters of enriched GO biological processes predicted to be stimulated by miRNA downregulation in senescent IMR90 fibroblasts.

Cluster #	GO biological processes	Count^2^	P-Value^3^	MiRNAs^4^
**Cluster 1** (ES = 3.5)^1^	GO:0045785∼positive regulation of cell adhesion	5	0.001	let-7b; LNA_let-7b; hsa-miR-1; 30a-3p; 34a; 124; 153; 155; 194; 203; 221; 222; 483-5p; 491-3p; 492; 495; 573; 584; 640; 891a
	GO:0030155∼regulation of cell adhesion	6	0.004	
	GO:0022407∼regulation of cell-cell adhesion	3	0.012	
**Cluster 2** (ES = 3.4)	GO:0043412∼biopolymer modification	25	0.000	let-7b; LNA_let-7b; hsa-miR-1; 26b; 27a; 27b; 29a; 29b; 29c; 30a-3p; 34a; 124; 138; 145; 153; 155; 181a; 182; 183; 212
	GO:0044267∼cellular protein metabolic process	29	0.011	
	GO:0019538∼protein metabolic process	31	0.034	
**Cluster 3** (ES = 2.8)	GO:0007050∼cell cycle arrest	5	0.008	let-7b; LNA_let-7b; hsa-miR-1; 15a; 15b; 19b; 21; 24; 27a; 30a-3p; 96; 130a; 132; 153; 182; 183; 186; 203; 212; 217
	GO:0043067∼regulation of programmed cell death	14	0.010	
	GO:0043066∼negative regulation of apoptosis	8	0.020	
	GO:0043069∼negative regulation of programmed cell death	8	0.021	
	GO:0060548∼negative regulation of cell death	8	0.022	
**Cluster 4** (ES = 2.6)	GO:0043434∼response to peptide hormone stimulus	6	0.007	hsa-miR-1; 16; 21; 27a; 27b; 29a; 29b; 29c; 30b; 30c; 101; 132; 145; 153; 182; 206; 212; 214; 503; 549
**Cluster 5** (ES = 2.1)	GO:0016477∼cell migration	7	0.020	hsa-miR-1; 19a; 19b; 27b; 29a; 29b; 29c; 30a-3p; 34b; 124; 138; 155; 183; 200b; 200c; 218; 369-3p; 582-3p
	GO:0048870∼cell motility	7	0.032	

1 An enrichment score (ES) of 1.3 is equivalent to a non-log scale value of 0.05.

2 The gene members, which belong to an annotation term.

3 Fisher Exact p-value representing the degree of enrichment of the GO term.

4 The miRNAs predicted to regulate the biological processes in the corresponding functional cluster. We show the top 20 miRNAs with highest ExprTarget prediction scores.

**Table 4:** Functional annotation clusters of enriched GO biological processes predicted to be regulated by miRNA changes in senescent IMR90 and MRC5 fibroblasts.

GO biological processes predicted to be suppressed by miRNA upregulation			
Cluster #	GO biological processes	Count^2^	P-Value^3^
**Cluster 1** (ES = 2.2)^1^	GO:0031325∼positive regulation of cellular metabolic process	19	0.0006
	GO:0080090∼regulation of primary metabolic process	45	0.0011
	GO:0031328∼positive regulation of cellular biosynthetic process	15	0.0025
	GO:0051171∼regulation of nitrogen compound metabolic process	38	0.0052
**Cluster 2** (ES = 2.0)	GO:0042127∼regulation of cell proliferation	16	0.0035
	GO:0008284∼positive regulation of cell proliferation	10	0.0103
**Cluster 3** (ES = 1.8)	GO:0030324∼lung development	5	0.0110
	GO:0030323∼respiratory tube development	5	0.0122
**Cluster 4** (ES = 1.7)	GO:0045595∼regulation of cell differentiation	11	0.0108
	GO:0045637∼regulation of myeloid cell differentiation	4	0.0232
**Cluster 5** (ES = 1.5)	GO:0045595∼regulation of cell differentiation	11	0.0108
	GO:0051094∼positive regulation of developmental process	7	0.0349
**Cluster 6** (ES = 1.4)	GO:0048513∼organ development	27	0.0037
	GO:0007517∼muscle organ development	7	0.0104
	GO:0007507∼heart development	7	0.0114
**Cluster 7** (ES = 1.3)	GO:0001944∼vasculature development	7	0.0227
	GO:0001568∼blood vessel development	7	0.0245

GO biological processes predicted to be stimulated by miRNA downregulation			
**Cluster 1** (ES = 1.3)	GO:0022409∼positive regulation of cell-cell adhesion	3	0.0003
	GO:0022407∼regulation of cell-cell adhesion	3	0.0011
**Cluster 2** (ES = 1.2)	GO:0043412∼biopolymer modification	9	0.0180

1,2,3 are the same as in Table 3.

To identify miRNAs that regulate the biological processes highlighted in the annotation clusters, genes associated with all GO terms within each annotation cluster were pooled and used to perform target-centric queries with the ExprTarget integrative prediction algorithm, specifying a score cutoff of 1. A gene list pooled from all GO terms within a given annotation cluster is much more comprehensive than genes selected from individual GO terms. MiRNAs with significant ExprTarget prediction scores (Tables 2 and 3) were considered to be key regulators of the biological processes that were altered during senescence.

## Discussion

To further extend understanding of the role of miRNAs in cell senescence, we used deep sequencing to interrogate the miRNA transcriptomes of young and senescent IMR90 fibroblasts. Even though microarrays have been used to profile miRNA expression in senescence [27], [32]–[34], the microarray technology suffers from limitations in sensitivity and specificity [35]–[37]. Deep sequencing technology overcomes the disadvantages of microarrays and generates millions of small RNA sequence reads, to measure absolute abundance and to discover novel microRNAs that have evaded previous discovery efforts. MiRDeep2 analysis of the sequencing reads detected 141 known miRNAs that were induced by senescence and 131 that were repressed, and discovered 20 novel miRNAs. Some of the novel miRNAs are differentially expressed between young and senescent fibroblasts, while still showing the characteristic pattern of higher expression of the mature miRNA over the star and loop sequences. These novel sequences were missed by traditional analyses because they tend to be expressed at low levels and they are located within unannotated regions of the genome. The low expression levels of novel miRNAs are effectively detected because of the high sensitivity of deep sequencing; low expression of novel miRNAs has been observed in other studies, suggesting that the more abundant miRNAs have largely already been discovered [38]. The targets and functions of the novel miRNAs remain to be investigated.

Among the known miRNAs detected in our study, members of the highly conserved let-7 family (let-7a, let-7f, let-7e, let-7i and let-7g) were the most abundant, with read counts up to 1.7 million and highly significant miRDeep2 detection scores. We found that senescence increased let-7 expression, as previously observed in microarray miRNA profiling studies [27]. Let-7 is among several miRNAs currently considered as tumor suppressors [39], [40], which is consistent with the view that senescence evolved as a tumor-suppressive mechanism to mitigate the hazard that cancer poses to longevity [5], [40]. Expression of let-7 is abnormal in 9 cancer types [41], [42]. Let-7 miRNA overexpression was shown to be associated with senescence in fibroids [43] and in skeletal muscle of aged humans, and was proposed to contribute to decreased muscle cell renewal and regeneration [44]. Taken together, these findings suggest that let-7 miRNAs play a key role in the control of cell senescence.

Members of the miR-449 family were among the most highly induced miRNAs in senescent fibroblasts. Consistent with a possible role of this miR-449 induction in cell cycle arrest during senescence, miR-449 has been shown to inhibit cell cycle progression at G1 phase by targeting CDK6 and CDC25A, which are pivotal to G1/S-phase transition [45]. Also, miR-499 was found to be significantly upregulated in senescent human mesenchymal stem cells, with the potential to regulate all four of the senescence induction types namely, telomere attrition, oxidative stress, oncogene expression and DNA damage signaling [20]. Among the miRNAs we found to be downregulated by senescence is the miR-17-92 cluster, which is a polycistron encoding 6 mature miRNAs (miR-17, -20a, 18a, -19a, -19b-1 and -92a-1). The expression of all 6 members was decreased in senescent cells with a substantial fold change (8.0, 7.8, 6.4, 10.8, 11.1, and 4.1, respectively). MiR-17-92 is overexpressed in human cancers, and promotes tumorigenesis mainly by inhibiting oncogene-induced senescence [46], [47]. Given the anti-senescence activity of miR-17-92, our finding that all miR-17-92 members are sharply repressed with senescence suggests that its very low levels may initiate and/or sustain the senescence program. Furthermore, miR-19b was found to be downregulated in several human replicative and organismal aging models [19], and has been identified as an oncogene that activates the AKT/mTOR pathway, which modulates organismal life spans [48].

miRNA microarray studies have reported four upregulated miRNAs (miR-152, -410, -431, and -493) and four downregulated miRNAs (miR-15a, -20a, -25, and -155) in both replicative and stress-induced senescence [28]. Decreased expression of one of these, miR-155, was observed in senescent BJ fibroblasts, aged primary human WI-38 fibroblasts, and peripheral blood mononuclear cells from older individuals [49]–[51]. In our study, all of these eight miRNAs were regulated in the same way. Another study reported twelve miRNAs as senescence regulators [20]. Four of twelve reported miRNAs (miR-217, -34a, -369-5p, and -20a) were regulated in the same way in our study; absence of the others in our study may reflect differences in cell types and senescence models. Furthermore, miR-217 has been reported to play a role in endothelial senescence and is implicated in the pathogenesis of atherosclerosis [52]. The expression of miR-217 increases in aged endothelial cells and promotes senescence through inhibition of SirT1, which is known to promote longevity and mediates the beneficial effects of calorie restriction.

Our study identified many miRNAs that are reported here for the first time as differentially regulated by senescence, adding to the increasing evidence for miRNA regulation of the senescent program. Mir-432 is highly induced by senescence in our data as illustrated in Fig. 2A, but was never reported in previous cell senescence microarray studies. It is mentioned in the literature only as abundant in the earliest stage of fetal development [53], and has a putative binding site in the 3′ UTR of some endothelial cell-restricted genes [54]. Other miRNAs found in our study to be affected during senescence (Fig. 3), but not reported in previous senescence studies, include miR-1246, miR-584 and miR-323, which are implicated in certain cancers [55]–[57]. Further investigation of these miRNAs may shed new light on the roles and mechanisms of miRNAs in cellular senescence.

To improve the identification of target genes of the miRNAs that changed expression during senescence, we adopted an approach based on co-analysis of changes in both miRNA and mRNA expressions. The mRNA transcripts that were detected by microarrays as differentially expressed during senescence, and simultaneously predicted *in silico* as targets of differentially expressed miRNAs, were considered as potential target genes regulated by senescence-induced miRNA changes. Matching the *in silico* predicted target genes with the differentially regulated mRNA transcripts derived from microarrays may minimize the false positives and negatives obtained from *in silico* prediction. In total, 386 genes were predicted to be the targets of up-regulated miRNAs in senescent cells, while 131 genes were predicted as targets of down-regulated miRNAs (Tables S3 and S4). We also included another fibroblast type, MRC5, in the matching analysis. We found 143 genes potentially downregulated by senescence-induced miRNA overexpression in both types of fibroblasts, IMR90 and MRC5, and 36 genes potentially upregulated by senescence-induced miRNA underexpression (Tables S5 and S6). To establish the impact of these miRNA targets on senescence, we used functional annotation to identify the most relevant and meaningful biological processes and pathways associated with these potential target genes. Terms related to positive regulation of cell proliferation are by far the most highly represented and enriched among the target genes suppressed by miRNA upregulation during senescence. This is consistent with a pivotal role for miRNAs in senescence regulation, since inhibition of proliferation-promoting genes is the hallmark of senescence [5]. In parallel to the inhibition of cell proliferation during senescence, we found that miRNAs tend to promote negative regulation of apoptosis; terms such as ‘negative regulation of apoptosis’ and ‘negative regulation of programmed cell death’ were significantly enriched among the target genes stimulated by miRNA downregulation during senescence. The cell commitment to senescence instead of apoptosis is pathologically relevant because, while apoptosis eliminates damaged or stressed cells, senescence arrests their growth and allows damaged cells to persist and acquire abnormalities that alter tissue microenvironment and promote aging and cancer. Our data suggests that miRNAs may sway the cellular decision to commit to senescence instead of apoptosis. Senescent fibroblasts are known to acquire resistance to apoptotic stimuli [30], however, it remains unclear what makes a cell undergo senescence or apoptosis [5]. The miRNAs found here to be potential regulators of apoptosis resistance in senescent cells could be used to investigate the mechanisms responsible for committing cells to senescence instead of apoptosis.

Other enriched biological processes suppressed by miRNA upregulation during senescence include positive regulation of cellular metabolic and biosynthetic processes, indicating an inhibition of biosynthetic pathways, which may reflect the cessation of growth during senescence. However, GO terms related to protein metabolic processes and biopolymer modification were significantly enriched among the target genes stimulated by miRNA downregulation during senescence. The stimulation of protein metabolism and biopolymer modification is consistent with evidence that anabolic processes are enhanced during cellular senescence [31] and could be part of metabolic alterations responsible for the increase in cell volume and mass resulting in the enlarged and flattened cell morphology that is typical of senescence. Furthermore, the KEGG pathway ‘actin cytoskeleton’, which is important for cell morphology, was also significantly enriched (Figure S1); its dysregulation may be involved in rearranging cytoskeletal structures to give rise to the flattened cell morphology. We also found that terms related to positive regulation of cell adhesion were significantly enriched among the target genes stimulated by miRNA downregulation during senescence. Since the capability of cells to adhere to each other and to the extracellular matrix is linked to cytoskeleton organization and cellular shape, the changes in cell adhesion regulation may contribute to the morphological changes induced by senescence. In support of our result, a previous functional study of senescence-associated miRNA targets showed enrichment in pathways involved in cytoskeletal remodeling [20]. Thus, miRNAs may regulate the morphological changes characteristic of the senescence phenotype.

In conclusion, our deep sequencing study has identified many known miRNAs that are differentially regulated with IMR90 cell senescence, and a number of novel miRNAs. Our results included known miRNAs that have not been previously reported as differentially regulated by senescence in microarray profiling studies, thus adding to the increasing evidence for miRNA contribution to senescence. Functional analysis of mRNA transcripts targeted by senescence-regulated miRNAs indicates that miRNAs may be key contributors to the cellular changes that make up the senescence phenotype. Hence, our findings could be used to generate new hypotheses to be tested in follow-up studies designed to further elucidate the role of miRNAs in senescence.

## Materials and Methods

### Cell culture

IMR90 fetal lung fibroblasts were purchased from ATCC (CCL-186) and maintained as described [58]. Briefly, cells were grown in DMEM supplemented with 10% FBS, and maintained at 37°C in 5% CO_2_. Cells were serially passaged and were considered senescent when they stopped dividing for 2 weeks. In this study “young” IMR90 cells are cells grown for less than 14 passagings; senescent cells were passaged more than 34 times, and had ceased further growth.

### RNA extraction and small RNA library construction

Total RNA, including small RNAs, was extracted from young and senescent IMR90 cells with the QIAGEN miRNeasy Mini Kit. Integrity of total RNA was checked with an Agilent 2100 Bioanalyzer. A total of 10 µg of small-RNA-enriched total RNA was electrophoresed on an 18% denaturing polyacrylamide gel with NEB miRNA markers, the gel stained with SYBR Gold, and the region corresponding to the 18–30 nucleotide bands in the marker lane was excised. Small RNA was eluted from the gel fragment and purified by standard methods. Illumina libraries were constructed from RNA specimens using the Illumina Small RNA Library kit, following the manufacturer's protocol. Briefly, 5′ and 3′ adapters were ligated to gel-purified small RNA in two separate steps, each followed by acrylamide gel purification. The ligation products were used for cDNA synthesis, followed by acrylamide gel purification and a final step of PCR amplification to generate libraries. One µl of each library was loaded on an Agilent Technologies 2100 Bioanalyzer to check size, purity, and concentration. Libraries were sequenced on an Illumina GA*_II_* instrument to generate 36 base reads. Sequencing data was processed with the Illumina pipeline v1.3.2. The MIAME-compliant sequencing data has been deposited in Gene Expression Omnibus (GEO) with accession number GSE27404.

### MiRDeep2 analysis of sequencing reads

Raw small RNA sequencing data were analyzed with miRDeep2 [59], a probabilistic algorithm based on the miRNA biogenesis model and designed to detect miRNAs from deep sequencing reads. Briefly, miRDeep2 pre-processed raw sequencing reads by removing the 3′ adapter sequence and discarding reads shorter than 18 nucleotides, before aligning reads to the human genome (NCBI36/hg18). Only reads that mapped perfectly to the genome five or less times were used for miRNA detection, since human miRNAs usually map to few genomic locations. MiRDeep2 estimates expression levels of known miRNAs, and also identifies novel miRNAs. The situation with deep sequencing is quite different from a microarray experiment, where only known miRNAs appear as features: any genomic sequence may potentially appear in the sequence dataset, and the informatics task is to determine which of these are miRNAs. Small RNAs identified by short read sequencing are derived from longer RNAs, and may or may not be true miRNAs. The miRDeep2 algorithm is based on the miRNA biogenesis model; it aligns reads to potential hairpin structures in a manner consistent with Dicer processing and assigns scores that measure the probability that hairpins are true miRNA precursors. MiRDeep2 then uses known characteristics of miRNA biogenesis to score the likelihood that the reads are derived from true miRNAs: a small RNA derived from the 5′ end of a predicted precursor is likely to be a miRNA if it is highly abundant relative to small RNAs derived from loop and star regions of the precursor, and less likely to be a true miRNA if it is present in similar proportions to the loop and star. The miRDeep2 algorithm uses this principle to produce a log-odds score that a small RNA is a true miRNA; it outputs a scored list of known and novel miRNAs as well as their expression levels.

### Statistical analysis of differential miRNA expression

In addition to identifying mature miRNAs in deep sequenced small RNA samples, miRDeep2 also generates expression values for the detected miRNAs. To test for differential miRNA expression between young and senescent fibroblasts, expression data for known miRNAs produced by miRDeep2 was used as input for the Bioconductor DESeq package [26]. DESeq uses a negative binomial distribution model to test for differential expression in deep sequencing datasets. The list of differentially expressed miRNAs produced by DESeq was further filtered to remove miRNAs with less than 10 reads in both samples, and fold change between samples less than 1.5.

### Potential target genes regulated by senescence-induced miRNA expression changes

To identify mRNA expression patterns associated with miRNA expression changes, we matched miRNA and mRNA expression data, and considered a miRNA to be regulatory only if expression levels of miRNA and its known mRNA targets are anti-correlated. This analysis was carried out in 3 steps:

#### Step 1. *In silico* prediction of genes targeted by miRNAs displaying differential expression in senescent fibroblasts

To functionally characterize the differentially expressed miRNAs, we identified their target transcripts, using ExprTargetDB [29], which uses a public database of human miRNA targets, to yield a comprehensive catalogue of miRNA targets. We queried ExprTargetDB with the 272 miRNAs displaying differential expression in senescent IMR90 fibroblasts (listed in Table S4) by selecting TargetScan as prediction algorithm and specifying a p-value cutoff of 0.05. Since we expect an inverse relationship between mRNA and miRNA expression, we predicted the transcripts targeted by upregulated miRNAs separately from the ones targeted by downregulated miRNAs. This analysis yielded two lists of upregulated and downregulated mRNA transcripts that are potentially regulated by senescence-induced changes in miRNA expression.

#### Step 2. Gene expression profiling of senescence

MIAME-compliant raw data of mRNA microarrays (Affymetrix Human Genome U133 Plus 2.0; containing 39,000 genes) were obtained from the Gene Expression Omnibus repository (accession number GSE19018). The CEL files GSM470491, GSM470492, and GSM470493 were from young and GSM470494, GSM470495, and GSM470496 from senescent IMR90 human fibroblasts. We used Robust Multichip Average (RMA) algorithms of the Bioconductor R package affy [60], [61] to pre-process the raw CEL files and normalize the probe expression levels. Normalized data were tested for differential expression with the SAM module in the Bioconductor siggenes package (http://bioconductor.org; [62]) using a cut-off of 15% FDR. A second set of MIAME-compliant raw data of mRNA two-color microarrays (NCI/ATC Hs-OperonV2; featuring 21,329 probes) was obtained from the Gene Expression Omnibus repository (accession number GSE15919; [63]. This set contains 6 arrays, which were hybridized with RNAs from MRC5 fibroblasts at population doubling 63 (senescent) and from MRC5 fibroblasts at population doubling 28 (young). The six arrays correspond to 3 biological replicates, each with a dye-swap technical replicate. The arrays were analyzed with Bioconductor limma package [64], [65] to identify genes differentially expressed in senescent MRC5 fibroblasts. Limma uses linear models to analyze microarray experiments. Briefly, the intensity data were imported with a filter so that any spot with a flag of −50 or less gets zero weight. The flag function assigns a zero to a normal spot and increasingly negative values for increasingly problematic spots. We implemented the ‘normexp’ method with an offset of 50 to correct the background and a loess normalization method (‘printtiploess’) to normalize within arrays. Differentially expressed genes were obtained by fitting a linear model to the normalized data followed by computing empirical Bayes statistics. The dye-effect was included in the model to adjust for any probe-specific dye effects. The p-values were adjusted for multiple testing using Benjamini and Hochberg's method to control the false discovery rate (FDR). Genes with FDR below 0.05 were selected as differentially expressed. The differentially expressed genes were separated into 2 lists of upregulated and downregulated genes, which we used in the next step of the analysis.

#### Step 3. Identification of potential target mRNAs by matching the *in silico* predicted miRNA target genes to experimentally determined microarray mRNA expression

We identified potential gene targets of the upregulated miRNAs by comparison of their predicted target mRNAs (from step 1) with downregulated mRNAs derived from the senescent IMR90 or MRC5 microarray studies (from step 2). Similarly, predicted target mRNAs for downregulated miRNAs were compared to upregulated mRNAs derived from the microarray study. These genes resulting from matching analysis of computational prediction of miRNA target genes and differentially expressed genes in microarray senescence experiments were considered as genes potentially regulated by senescence-induced miRNA changes, and were chosen for downstream functional and pathway analyses.

### Functional annotation of miRNA targets

To characterize biological processes affected by senescence-induced miRNA expression changes, we used Gene Ontology (GO) and the functional annotation clustering feature of DAVID [66], [67] to functionally annotate genes that are potentially regulated by miRNAs during senescence. We also used the clustering analysis to analyze the significance of KEGG pathways in our data. The functional annotation clustering tool measures the similarities among GO terms based on the extent of their associated genes and assembles the similar and redundant GO terms into annotation clusters. Each GO term in a cluster is assigned a Fisher Exact p-value representing the degree of enrichment of the GO term in the input gene list. Each cluster is assigned an enrichment score to rank its biological significance. This enrichment score is derived from the geometric mean (in -log scale) of member's p-values. Thus, a biologically significant cluster (high enrichment score) is generated only when most of its GO term members have significant enrichment values (low Fisher Exact p-values). The resulting clusters were further curated to keep only GO terms with p-values <0.05.

### Identification of miRNAs associated with biological processes altered by senescence

To identify miRNAs that are key regulators of the biological processes included in the annotation clusters, the GO terms within each annotation cluster were subjected to computational analysis. Genes associated with all GO terms within each annotation cluster were retrieved using DAVID's genes link (G) provided for each cluster. This function pools all genes from different GO terms within a selected cluster into one list. Genes pooled from each annotation cluster were submitted to the public database of human miRNA targets (ExprTargetDB) to perform target-centric queries using the ExprTarget integrative prediction algorithm. MiRNAs resulting from this analysis were further filtered to keep only miRNAs with significant prediction scores. These miRNAs are predicted to be key regulators of the biological processes that are altered during senescence.

## Supporting Information

Figure S1 Significantly overrepresented KEGG ‘actin cytoskeleton’ pathway associated with miRNAs overexpressed during senescence. KEGG pathway mapping was performed using DAVID. The genes marked with an oval represent the targets of the senescence-induced miRNA overexpression.
(TIFF)

Table S1 Survey of miRDeep2 performance showing number of novel and known miRNAs and value of signal-to-noise ratio under different score cut-offs ranging from 10 to 1.
(DOC)

Table S2 MiRNAs differentially expressed in cell senescence.
(DOC)

Table S3 Potential target genes downregulated by senescence-induced miRNA overexpression in IMR90 fibroblasts.
(DOC)

Table S4 Potential target genes upregulated by senescence-induced miRNA underexpression in IMR90 fibroblasts.
(DOC)

Table S5 Potential target genes downregulated by senescence-induced miRNA overexpression in both types of fibroblasts, IMR90 and MRC5.
(DOC)

Table S6 Potential target genes upregulated by senescence-induced miRNA underexpression in both types of fibroblasts, IMR90 and MRC5.
(DOC)
