# Supplementary figures and images for: Deep Sequencing Reveals Novel MicroRNAs and Regulation of MicroRNA Expression during Cell Senescence
Source: PLoS One. 2011 May 26;6(5):e20509. doi: 10.1371/journal.pone.0020509 (PMC3102725; doi:10.1371/journal.pone.0020509)

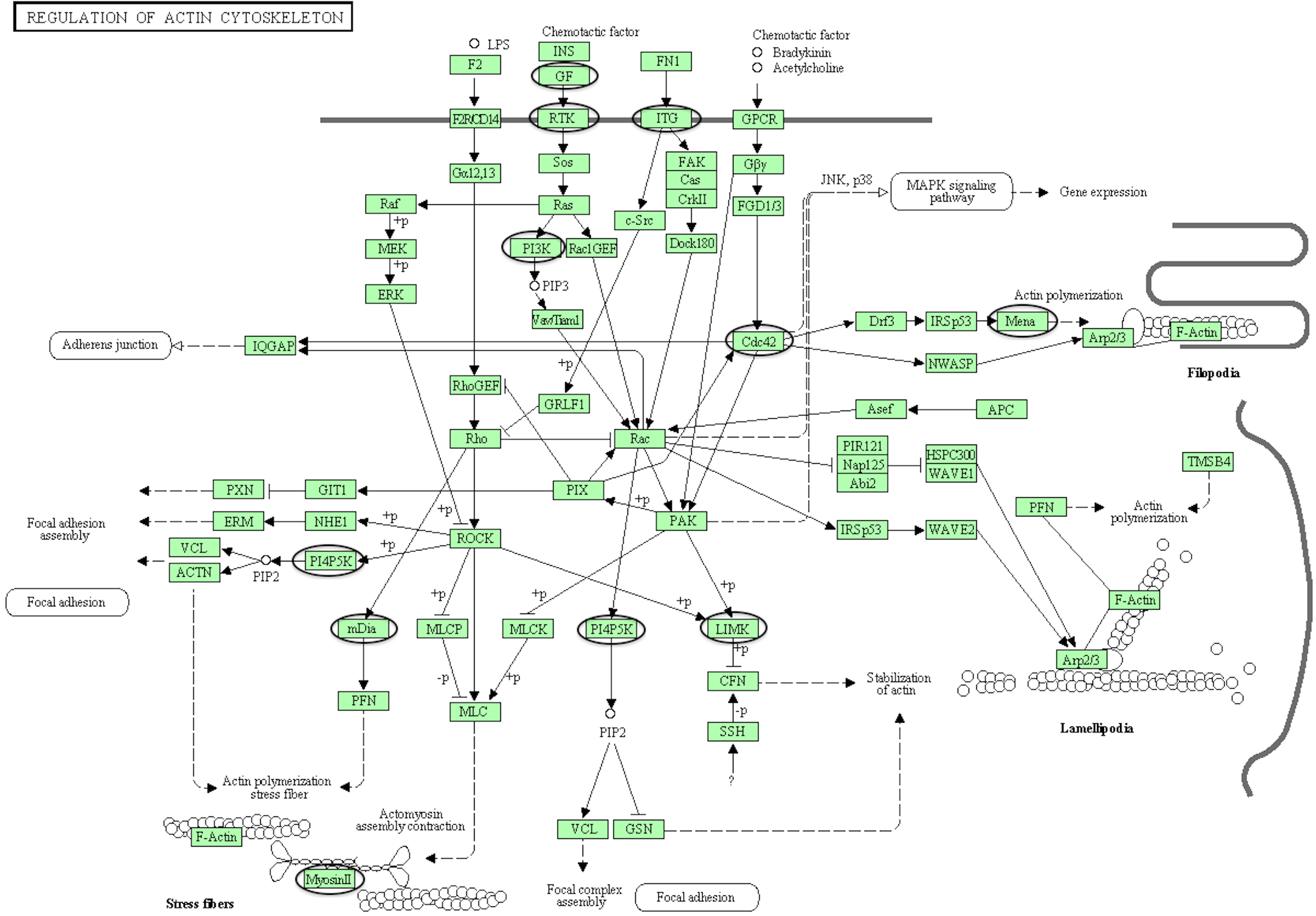

Supplement: Figure S1 — Significantly overrepresented KEGG ‘actin cytoskeleton’ pathway associated with miRNAs overexpressed during senescence. KEGG pathway mapping was performed using DAVID. The genes marked with an oval represent the targets of the senescence-induced miRNA overexpression. (TIFF) [file pone.0020509.s001.tif]
