# Supplementary material for: Deep Sequencing Reveals Novel MicroRNAs and Regulation of MicroRNA Expression during Cell Senescence
Source: PLoS One. 2011 May 26;6(5):e20509. doi: 10.1371/journal.pone.0020509 (PMC3102725; doi:10.1371/journal.pone.0020509)
Supplement: Table S1 — Survey of miRDeep2 performance showing number of novel and known miRNAs and value of signal-to-noise ratio under different score cut-offs ranging from 10 to 1. (DOC) [file pone.0020509.s002.doc]

**Table S1**. Survey of miRDeep2 performance showing the number of novel and known miRNAs and value of signal-to-noise ratio under different score cut-offs ranging from 10 to 1.

|  | **Novel miRNAs** | | |  | **Known miRNAs** | | |  |
| --- | --- | --- | --- | --- | --- | --- | --- | --- |
| **miRDeep2 score^1^** | **Predicted^2^** | **False positives^3^** | **True positives^4^** |  | **In species^5^** | **In data^6^** | **Detected^7^** | **Signal-to-noise^8^** |
| 10 | 27 | 8 ± 3 | 19 ± 3 (72 ± 10%) |  | 1223 | 656 | 358 (55%) | 23.3 |
| 9 | 28 | 8 ± 3 | 20 ± 3 (72 ± 9%) |  | 1223 | 656 | 360 (55%) | 23 |
| 8 | 31 | 8 ± 3 | 23 ± 3 (74 ± 9%) |  | 1223 | 656 | 365 (56%) | 23 |
| 7 | 32 | 8 ± 3 | 24 ± 3 (75 ± 9%) |  | 1223 | 656 | 369 (56%) | 23 |
| 6 | 35 | 8 ± 3 | 27 ± 3 (76 ± 8%) |  | 1223 | 656 | 373 (57%) | 22.7 |
| 5 | 39 | 10 ± 3 | 29 ± 3 (75 ± 8%) |  | 1223 | 656 | 432 (66%) | 21.5 |
| 4 | 46 | 15 ± 4 | 31 ± 4 (66 ± 8%) |  | 1223 | 656 | 452 (69%) | 15.6 |
| 3 | 53 | 33 ± 5 | 20 ± 5 (37 ± 10%) |  | 1223 | 656 | 465 (71%) | 8.6 |
| 2 | 78 | 45 ± 6 | 33 ± 6 (43 ± 8%) |  | 1223 | 656 | 485 (74%) | 7.1 |
| 1 | 152 | 63 ± 7 | 89 ± 7 (59 ± 5%) |  | 1223 | 656 | 528 (80%) | 6 |

^1^The miRDeep2 score represents the log-odds probability of a sequence being genuine miRNA precursor versus the probability that it is a background hairpin, given the evidence from the data.

^2^Number of novel miRNA hairpins with a score ≥ cut-off.

^3^Number of false positive miRNA hairpins predicted at this cut-off, as estimated by the miRDeep2 controls. Mean and standard deviation are estimated from 100 rounds of permuted controls.

^4^Number of true positive miRNA hairpins is estimated as t = total novel miRNAs - false positive novel miRNAs. The percentage of the predicted novel miRNAs that is estimated to be true positives is calculated as p = t/total novel miRNAs. The number of false positives is estimated from 100 rounds of permuted controls. In each of the 100 rounds, t and p are calculated, generating mean and standard deviation of t and p. The variable p can be used as an estimation of miRDeep2 positive predictive value at the score cut-off.

^5^Number of reference mature miRNAs for the human species given as input to miRDeep2.

^6^Number of reference mature miRNAs that map perfectly to one or more of precursor candidates that have been excised from the genome by miRDeep2.

^7^Number of reference mature miRNAs that map perfectly to one or more of predicted miRNA hairpins that have a score equal to or exceeding the cut-off. The percentage of reference mature miRNAs in data that is detected by miRDeep2 is calculated as s = reference mature miRNAs detected/reference mature miRNAs in data. s can be used as an estimation of miRDeep2 sensitivity at the score cut-off.

^8^The signal-to-noise ratio for the given score cut-off is estimated as r = total miRNA hairpins reported / mean estimated false positive miRNA hairpins over 100 rounds of permuted controls.
