# Supplementary material for: Deep Sequencing Reveals Novel MicroRNAs and Regulation of MicroRNA Expression during Cell Senescence
Source: PLoS One. 2011 May 26;6(5):e20509. doi: 10.1371/journal.pone.0020509 (PMC3102725; doi:10.1371/journal.pone.0020509)
Supplement: Table S2 — MiRNAs differentially expressed in cell senescence. (DOC) [file pone.0020509.s003.doc]

**Table S2**. miRNAs differentially expressed during cell senescence.

| **Upregulated** | |  | **Downregulated** | |
| --- | --- | --- | --- | --- |
| **miRNA** | **Fold Change*** |  | **miRNA** | **Fold Change*** |
| hsa-miR-449a | 194.2 |  | hsa-miR-199b-5p | -37.6 |
| hsa-miR-449c | 157.6 |  | hsa-miR-549 | -26.8 |
| hsa-miR-129-5p | 25.7 |  | hsa-miR-551b | -26.5 |
| hsa-miR-765 | 15.3 |  | hsa-miR-1245 | -23.7 |
| hsa-miR-375 | 14.4 |  | hsa-miR-766 | -22.2 |
| hsa-miR-184 | 13.8 |  | hsa-miR-296-3p | -20.5 |
| hsa-miR-1290 | 13.8 |  | hsa-miR-33b | -15.5 |
| hsa-miR-3656 | 11.1 |  | hsa-miR-218 | -14.9 |
| hsa-miR-126 | 9.2 |  | hsa-miR-3622a-5p | -13.4 |
| hsa-miR-217 | 8.7 |  | hsa-miR-145 | -12.3 |
| hsa-miR-1246 | 8.3 |  | hsa-miR-146b-3p | -12.0 |
| hsa-miR-129-3p | 8.2 |  | hsa-miR-497 | -11.7 |
| hsa-miR-432 | 7.5 |  | hsa-miR-19b | -11.1 |
| hsa-miR-1197 | 7.2 |  | hsa-miR-19a | -10.8 |
| hsa-miR-584 | 6.9 |  | hsa-miR-1270 | -10.7 |
| hsa-miR-1274a | 6.9 |  | hsa-miR-548u | -9.9 |
| hsa-miR-1275 | 6.9 |  | hsa-miR-15a | -9.0 |
| hsa-miR-449b | 5.9 |  | hsa-miR-155 | -8.5 |
| hsa-miR-3911 | 5.6 |  | hsa-miR-16 | -8.4 |
| hsa-miR-1274b | 5.4 |  | hsa-miR-3912 | -8.4 |
| hsa-miR-122 | 5.1 |  | hsa-miR-576-5p | -8.4 |
| hsa-miR-668 | 5.1 |  | hsa-miR-3154 | -8.2 |
| hsa-miR-323b-3p | 4.8 |  | hsa-miR-17 | -8.0 |
| hsa-miR-491-5p | 4.7 |  | hsa-miR-15b | -8.0 |
| hsa-miR-539 | 4.6 |  | hsa-miR-20a | -7.8 |
| hsa-miR-496 | 4.6 |  | hsa-miR-148a | -7.2 |
| hsa-miR-541 | 4.4 |  | hsa-miR-106a | -7.2 |
| hsa-miR-483-5p | 4.3 |  | hsa-miR-18a | -6.4 |
| hsa-miR-323b-5p | 4.3 |  | hsa-miR-1261 | -5.8 |
| hsa-miR-1293 | 4.2 |  | hsa-miR-196a | -5.6 |
| hsa-miR-664 | 4.2 |  | hsa-miR-1271 | -5.6 |
| hsa-miR-1268 | 4.2 |  | hsa-miR-296-5p | -5.5 |
| hsa-miR-654-5p | 4.2 |  | hsa-miR-887 | -5.4 |
| hsa-miR-210 | 4.1 |  | hsa-miR-18b | -5.2 |
| hsa-miR-543 | 4.1 |  | hsa-miR-345 | -5.0 |
| hsa-miR-628-5p | 3.9 |  | hsa-miR-195 | -5.0 |
| hsa-miR-3180 | 3.8 |  | hsa-miR-3187 | -4.6 |
| hsa-miR-3180-3p | 3.8 |  | hsa-miR-146a | -4.6 |
| hsa-miR-330-5p | 3.7 |  | hsa-miR-490-5p | -4.5 |
| hsa-miR-30e | 3.6 |  | hsa-miR-106b | -4.4 |
| hsa-miR-323-5p | 3.6 |  | hsa-miR-34c-5p | -4.4 |
| hsa-miR-369-3p | 3.5 |  | hsa-miR-1302 | -4.2 |
| hsa-miR-935 | 3.3 |  | hsa-miR-548z | -4.2 |
| hsa-miR-323-3p | 3.2 |  | hsa-miR-196b | -4.2 |
| hsa-miR-3909 | 3.1 |  | hsa-miR-92a | -4.1 |
| hsa-miR-337-3p | 3.1 |  | hsa-miR-490-3p | -4.0 |
| hsa-miR-889 | 2.9 |  | hsa-miR-33a | -3.9 |
| hsa-miR-137 | 2.9 |  | hsa-miR-3170 | -3.8 |
| hsa-miR-874 | 2.9 |  | hsa-miR-7 | -3.8 |
| hsa-miR-185 | 2.9 |  | hsa-miR-3158 | -3.7 |
| hsa-miR-493 | 2.9 |  | hsa-miR-93 | -3.7 |
| hsa-miR-589 | 2.8 |  | hsa-miR-1255a | -3.5 |
| hsa-miR-224 | 2.8 |  | hsa-miR-3130-3p | -3.1 |
| hsa-miR-758 | 2.8 |  | hsa-miR-1254 | -3.1 |
| hsa-miR-410 | 2.8 |  | hsa-miR-641 | -3.1 |
| hsa-miR-127-3p | 2.7 |  | hsa-miR-671-5p | -3.1 |
| hsa-miR-656 | 2.6 |  | hsa-miR-629 | -3.1 |
| hsa-miR-30a | 2.6 |  | hsa-miR-505 | -3.1 |
| hsa-miR-23c | 2.6 |  | hsa-miR-3913 | -3.1 |
| hsa-miR-30d | 2.6 |  | hsa-miR-548p | -3.1 |
| hsa-miR-24 | 2.6 |  | hsa-miR-424 | -3.0 |
| hsa-miR-494 | 2.5 |  | hsa-miR-548k | -3.0 |
| hsa-miR-1294 | 2.5 |  | hsa-miR-3129 | -2.9 |
| hsa-miR-27a | 2.5 |  | hsa-miR-146b-5p | -2.9 |
| hsa-miR-495 | 2.5 |  | hsa-miR-548a-3p | -2.8 |
| hsa-miR-379 | 2.4 |  | hsa-miR-1296 | -2.8 |
| hsa-miR-98 | 2.4 |  | hsa-miR-3164 | -2.8 |
| hsa-miR-2110 | 2.4 |  | hsa-miR-576-3p | -2.8 |
| hsa-miR-1307 | 2.3 |  | hsa-miR-25 | -2.8 |
| hsa-miR-370 | 2.3 |  | hsa-miR-3928 | -2.8 |
| hsa-miR-23a | 2.3 |  | hsa-miR-383 | -2.8 |
| hsa-miR-23b | 2.3 |  | hsa-miR-3151 | -2.8 |
| hsa-miR-125a-5p | 2.3 |  | hsa-miR-3200-5p | -2.7 |
| hsa-miR-326 | 2.3 |  | hsa-miR-598 | -2.6 |
| hsa-miR-409-5p | 2.3 |  | hsa-miR-130b | -2.6 |
| hsa-miR-27b | 2.3 |  | hsa-miR-652 | -2.6 |
| hsa-miR-485-5p | 2.2 |  | hsa-miR-378b | -2.5 |
| hsa-miR-660 | 2.2 |  | hsa-miR-3174 | -2.4 |
| hsa-miR-128 | 2.2 |  | hsa-miR-1908 | -2.4 |
| hsa-miR-487b | 2.1 |  | hsa-miR-3136 | -2.4 |
| hsa-miR-486-5p | 2.1 |  | hsa-miR-143 | -2.4 |
| hsa-miR-369-5p | 2.1 |  | hsa-miR-342-5p | -2.4 |
| hsa-miR-433 | 2.1 |  | hsa-miR-342-3p | -2.3 |
| hsa-miR-1273d | 2.1 |  | hsa-miR-942 | -2.3 |
| hsa-miR-501-3p | 2.0 |  | hsa-miR-190 | -2.3 |
| hsa-miR-3605-5p | 2.0 |  | hsa-miR-548e | -2.3 |
| hsa-miR-31 | 2.0 |  | hsa-miR-1278 | -2.2 |
| hsa-miR-222 | 2.0 |  | hsa-miR-1273c | -2.2 |
| hsa-miR-193a-5p | 2.0 |  | hsa-miR-199a-3p | -2.2 |
| hsa-miR-3679-5p | 2.0 |  | hsa-miR-199b-3p | -2.2 |
| hsa-miR-1185 | 2.0 |  | hsa-miR-4286 | -2.2 |
| hsa-miR-181b | 2.0 |  | hsa-miR-548b-5p | -2.1 |
| hsa-miR-221 | 1.9 |  | hsa-miR-3127 | -2.1 |
| hsa-miR-532-3p | 1.9 |  | hsa-miR-331-5p | -2.1 |
| hsa-miR-502-3p | 1.9 |  | hsa-miR-219-1-3p | -2.1 |
| hsa-miR-134 | 1.9 |  | hsa-miR-708 | -2.1 |
| hsa-miR-125b | 1.9 |  | hsa-miR-625 | -2.1 |
| hsa-miR-181d | 1.9 |  | hsa-miR-720 | -2.0 |
| hsa-miR-329 | 1.8 |  | hsa-miR-425 | -2.0 |
| hsa-miR-320e | 1.8 |  | hsa-miR-1256 | -2.0 |
| hsa-miR-431 | 1.8 |  | hsa-miR-3145 | -2.0 |
| hsa-miR-411 | 1.8 |  | hsa-miR-548n | -2.0 |
| hsa-let-7i | 1.8 |  | hsa-miR-455-3p | -1.9 |
| hsa-miR-1301 | 1.8 |  | hsa-miR-3615 | -1.9 |
| hsa-miR-3177 | 1.7 |  | hsa-miR-1262 | -1.9 |
| hsa-miR-320d | 1.7 |  | hsa-miR-301b | -1.9 |
| hsa-miR-487a | 1.7 |  | hsa-miR-301a | -1.8 |
| hsa-miR-3190 | 1.7 |  | hsa-miR-361-5p | -1.8 |
| hsa-miR-30c | 1.7 |  | hsa-miR-324-3p | -1.8 |
| hsa-miR-452 | 1.7 |  | hsa-miR-192 | -1.7 |
| hsa-miR-421 | 1.7 |  | hsa-miR-32 | -1.7 |
| hsa-let-7d | 1.7 |  | hsa-miR-212 | -1.7 |
| hsa-miR-34a | 1.7 |  | hsa-miR-454 | -1.7 |
| hsa-miR-380 | 1.7 |  | hsa-miR-503 | -1.7 |
| hsa-miR-486-3p | 1.7 |  | hsa-miR-542-5p | -1.7 |
| hsa-miR-769-5p | 1.6 |  | hsa-miR-215 | -1.7 |
| hsa-miR-127-5p | 1.6 |  | hsa-miR-194 | -1.7 |
| hsa-miR-654-3p | 1.6 |  | hsa-miR-423-3p | -1.7 |
| hsa-miR-328 | 1.6 |  | hsa-miR-1304 | -1.7 |
| hsa-miR-152 | 1.6 |  | hsa-miR-361-3p | -1.7 |
| hsa-miR-151-5p | 1.6 |  | hsa-miR-1260 | -1.6 |
| hsa-miR-149 | 1.6 |  | hsa-miR-193a-3p | -1.6 |
| hsa-miR-2355-3p | 1.6 |  | hsa-miR-374b | -1.6 |
| hsa-miR-3167 | 1.6 |  | hsa-miR-450b-5p | -1.6 |
| hsa-miR-382 | 1.6 |  | hsa-miR-1260b | -1.6 |
| hsa-miR-532-5p | 1.5 |  | hsa-miR-1285 | -1.5 |
| hsa-miR-3124 | 1.5 |  | hsa-miR-548o | -1.5 |
| hsa-miR-362-5p | 1.5 |  | hsa-miR-643 | -1.5 |
| hsa-miR-197 | 1.5 |  | hsa-miR-219-5p | -1.5 |
| hsa-miR-339-3p | 1.5 |  | hsa-miR-28-3p | -1.5 |
| hsa-miR-744 | 1.5 |  | hsa-miR-324-5p | -1.5 |
| hsa-let-7e | 1.5 |  | | |
| hsa-miR-665 | 1.5 |  | | |
| hsa-miR-154 | 1.5 |  | | |
| hsa-miR-409-3p | 1.5 |  | | |
| hsa-miR-148b | 1.5 |  | | |
| hsa-miR-377 | 1.5 |  | | |
| hsa-miR-378 | 1.5 |  | | |
| hsa-miR-181a | 1.5 |  | | |
| hsa-miR-548i | 1.5 |  | | |
| hsa-miR-299-5p | 1.5 |  | | |
