# Supplementary material for: Deep Sequencing Reveals Novel MicroRNAs and Regulation of MicroRNA Expression during Cell Senescence
Source: PLoS One. 2011 May 26;6(5):e20509. doi: 10.1371/journal.pone.0020509 (PMC3102725; doi:10.1371/journal.pone.0020509)
Supplement: Table S3 — Potential target genes downregulated by senescence-induced miRNA overexpression in IMR90 fibroblasts. (DOC) [file pone.0020509.s004.doc]

**Table S3. Potential target genes downregulated by senescence-induced miRNA overexpression in IMR90 fibroblasts.**

| **Affy id** | **Entrez gene id** | **Gene symbol** | **Gene name** | **Fold change*** |
| --- | --- | --- | --- | --- |
| 211756_at | 5744 | PTHLH | Parathyroid hormone-like hormone | -35.7 |
| 240509_s_at | 64388 | GREM2 | Gremlin 2, cysteine knot superfamily, homolog | -10.9 |
| 206115_at | 1960 | EGR3 | Early growth response 3 | -10.1 |
| 228574_at | 160335 | TMTC2 | Transmembrane and tetratricopeptide repeat containing 2 | -10.1 |
| 221898_at | 10630 | PDPN | Podoplanin | -9.4 |
| 204359_at | 23768 | FLRT2 | Fibronectin leucine rich transmembrane protein 2 | -6.0 |
| 203821_at | 1839 | HBEGF | Heparin-binding EGF-like growth factor | -5.6 |
| 203680_at | 5577 | PRKAR2B | Protein kinase, cAMP-dependent, regulatory, type II, beta | -5.6 |
| 212188_at | 115207 | KCTD12 | Potassium channel tetramerisation domain containing 12 | -5.3 |
| 214043_at | 5789 | PTPRD | Protein tyrosine phosphatase, receptor type, D | -5.2 |
| 227971_at | 203447 | NRK | Nik related kinase | -5.0 |
| 235579_at | 9169 | SFRS2IP | Splicing factor, arginine/serine-rich 2, interacting protein | -4.6 |
| 212670_at | 2006 | ELN | Elastin | -4.3 |
| 229572_at | 23545 | ATP6V0A2 | ATPase, H+ transporting, lysosomal V0 subunit a2 | -4.3 |
| 225056_at | 57568 | SIPA1L2 | Signal-induced proliferation-associated 1 like 2 | -4.3 |
| 212565_at | 23012 | STK38L | Serine/threonine kinase 38 like | -4.0 |
| 203184_at | 2201 | FBN2 | Fibrillin 2 | -4.0 |
| 238865_at | 132430 | PABPC4L | Poly(A) binding protein, cytoplasmic 4-like | -4.0 |
| 212327_at | 22998 | LIMCH1 | LIM and calponin homology domains 1 | -3.9 |
| 202668_at | 1948 | EFNB2 | Ephrin-B2 | -3.6 |
| 224840_at | 2289 | FKBP5 | FK506 binding protein 5 | -3.6 |
| 202948_at | 3554 | IL1R1 | Interleukin 1 receptor, type I | -3.4 |
| 217997_at | 22822 | PHLDA1 | Pleckstrin homology-like domain, family A, member 1 | -3.4 |
| 213712_at | 54898 | ELOVL2 | Elongation of very long chain fatty acids (FEN1/Elo2, SUR4/Elo3, yeast)-like 2 | -3.4 |
| 226534_at | 4254 | KITLG | KIT ligand | -3.4 |
| 1554822_at | 57157 | PHTF2 | Putative homeodomain transcription factor 2 | -3.4 |
| 203018_s_at | 117178 | SSX2IP | Synovial sarcoma, X breakpoint 2 interacting protein | -3.3 |
| 222450_at | 56937 | PMEPA1 | Prostate transmembrane protein, androgen induced 1 | -3.3 |
| 223092_at | 56172 | ANKH | Ankylosis, progressive homolog (mouse) | -3.3 |
| 227920_at | 57673 | BEND3 | BEN domain containing 3 | -3.3 |
| 212230_at | 8613 | PPAP2B | Phosphatidic acid phosphatase type 2B | -3.2 |
| 202817_s_at | 6760 | SS18 | Synovial sarcoma translocation, chromosome 18 | -3.2 |
| 228259_s_at | 64097 | EPB41L4A | Erythrocyte membrane protein band 4.1 like 4A | -3.2 |
| 228310_at | 55740 | ENAH | Enabled homolog (Drosophila) | -3.2 |
| 227231_at | 57482 | KIAA1211 | KIAA1211 | -3.1 |
| 213001_at | 23452 | ANGPTL2 | Angiopoietin-like 2 | -3.1 |
| 228915_at | 1602 | DACH1 | Dachshund homolog 1 (Drosophila) | -3.0 |
| 213310_at | 27161 | EIF2C2 | Eukaryotic translation initiation factor 2C, 2 | -3.0 |
| 228988_at | 7552 | ZNF711 | Zinc finger protein 711 | -3.0 |
| 226922_at | 5903 | RANBP2 | RAN binding protein 2 | -2.9 |
| 229057_at | 6326 | SCN2A | Sodium channel, voltage-gated, type II, alpha subunit | -2.9 |
| 213543_at | 6444 | SGCD | Sarcoglycan, delta (35kDa dystrophin-associated glycoprotein) | -2.9 |
| 212385_at | 6925 | TCF4 | Transcription factor 4 | -2.8 |
| 208051_s_at | 10605 | PAIP1 | Poly(A) binding protein interacting protein 1 | -2.8 |
| 209829_at | 9750 | FAM65B | Family with sequence similarity 65, member B | -2.8 |
| 236029_at | 120114 | FAT3 | FAT tumor suppressor homolog 3 (Drosophila) | -2.8 |
| 235711_at | 5814 | PURB | Purine-rich element binding protein B | -2.8 |
| 213438_at | 23114 | NFASC | Neurofascin homolog (chicken) | -2.8 |
| 235907_at | 55161 | TMEM33 | Transmembrane protein 33 | -2.7 |
| 219572_at | 93664 | CADPS2 | Ca++-dependent secretion activator 2 | -2.7 |
| 203579_s_at | 9057 | SLC7A6 | Solute carrier family 7 (cationic amino acid transporter, y+ system), member 6 | -2.7 |
| 201586_s_at | 6421 | SFPQ | Splicing factor proline/glutamine-rich | -2.7 |
| 221489_s_at | 81848 | SPRY4 | Sprouty homolog 4 (Drosophila) | -2.7 |
| 203432_at | 7112 | TMPO | Thymopoietin | -2.7 |
| 209588_at | 2048 | EPHB2 | EPH receptor B2 | -2.6 |
| 213067_at | 4628 | MYH10 | Myosin, heavy chain 10, non-muscle | -2.6 |
| 233970_s_at | 51605 | TRMT6 | tRNA methyltransferase 6 homolog (S. cerevisiae) | -2.6 |
| 202969_at | 8445 | DYRK2 | Dual-specificity tyrosine-(Y)-phosphorylation regulated kinase 2 | -2.6 |
| 204379_s_at | 2261 | FGFR3 | Fibroblast growth factor receptor 3 | -2.5 |
| 215305_at | 5156 | PDGFRA | Platelet-derived growth factor receptor, alpha polypeptide | -2.5 |
| 215323_at | 338645 | LUZP2 | Leucine zipper protein 2 | -2.5 |
| 202743_at | 8503 | PIK3R3 | Phosphoinositide-3-kinase, regulatory subunit 3 (gamma) | -2.5 |
| 225429_at | 5537 | PPP6C | Protein phosphatase 6, catalytic subunit | -2.4 |
| 213273_at | 26011 | ODZ4 | Odz, odd Oz/ten-m homolog 4 (Drosophila) | -2.4 |
| 214255_at | 57194 | ATP10A | ATPase, class V, type 10A | -2.4 |
| 226998_at | 80155 | NAA15 | NMDA receptor regulated 1 | -2.4 |
| 204700_x_at | 27042 | C1ORF107 | Chromosome 1 open reading frame 107 | -2.4 |
| 225354_s_at | 83699 | SH3BGRL2 | SH3 domain binding glutamic acid-rich protein like 2 | -2.4 |
| 235798_at | 100113407 | TMEM170B | Transmembrane protein 170B | -2.4 |
| 205100_at | 9945 | GFPT2 | Glutamine-fructose-6-phosphate transaminase 2 | -2.4 |
| 203881_s_at | 1756 | DMD | Dystrophin | -2.3 |
| 202679_at | 4864 | NPC1 | Niemann-Pick disease, type C1 | -2.3 |
| 213793_s_at | 9456 | HOMER1 | Homer homolog 1 (Drosophila) | -2.3 |
| 202674_s_at | 4008 | LMO7 | LIM domain 7 | -2.3 |
| 216268_s_at | 182 | JAG1 | Jagged 1 (Alagille syndrome) | -2.3 |
| 220386_s_at | 27436 | EML4 | Echinoderm microtubule associated protein like 4 | -2.3 |
| 227080_at | 90874 | ZNF697 | Hypothetical LOC100130667; zinc finger protein 697 | -2.3 |
| 225145_at | 57727 | NCOA5 | Nuclear receptor coactivator 5 | -2.3 |
| 213407_at | 23035 | PHLPP2 | PH domain and leucine rich repeat protein phosphatase 2 | -2.3 |
| 213725_x_at | 64131 | XYLT1 | Xylosyltransferase I | -2.3 |
| 1556361_s_at | 81573 | ANKRD13C | Ankyrin repeat domain 13C | -2.3 |
| 212239_at | 5295 | PIK3R1 | Phosphoinositide-3-kinase, regulatory subunit 1 (alpha) | -2.3 |
| 206108_s_at | 6431 | SFRS6 | Splicing factor, arginine/serine-rich 6; similar to arginine/serine-rich splicing factor 6 | -2.3 |
| 214113_s_at | 9939 | RBM8A | RNA binding motif protein 8A | -2.3 |
| 219470_x_at | 54619 | CCNJ | Cyclin J | -2.3 |
| 214155_s_at | 113251 | LARP4 | La ribonucleoprotein domain family, member 4 | -2.3 |
| 1555427_s_at | 10492 | SYNCRIP | Synaptotagmin binding, cytoplasmic RNA interacting protein | -2.2 |
| 234140_s_at | 57620 | STIM2 | Stromal interaction molecule 2 | -2.2 |
| 219367_s_at | 8828 | NRP2 | Neuropilin 2 | -2.2 |
| 225932_s_at | 3181 | HNRNPA2B1 | Heterogeneous nuclear ribonucleoprotein A2/B1 | -2.2 |
| 204225_at | 9759 | HDAC4 | Histone deacetylase 4 | -2.2 |
| 216466_at | 89795 | NAV3 | Neuron navigator 3; similar to neuron navigator 3 | -2.2 |
| 218871_x_at | 55454 | CSGALNACT2 | Chondroitin sulfate N-acetylgalactosaminyltransferase 2 | -2.2 |
| 238007_at | 10778 | ZNF271 | Zinc finger protein 271 | -2.2 |
| 224937_at | 5738 | PTGFRN | Prostaglandin F2 receptor negative regulator | -2.2 |
| 200644_at | 65108 | MARCKSL1 | MARCKS-like 1 | -2.2 |
| 212930_at | 490 | ATP2B1 | ATPase, Ca++ transporting, plasma membrane 1 | -2.1 |
| 213170_at | 2882 | GPX7 | Glutathione peroxidase 7 | -2.1 |
| 209406_at | 9532 | BAG2 | BCL2-associated athanogene 2 | -2.1 |
| 204337_at | 5999 | RGS4 | Regulator of G-protein signaling 4 | -2.1 |
| 204666_s_at | 80143 | SIKE1 | Suppressor of IKK epsilon | -2.1 |
| 231577_s_at | 2633 | GBP1 | Guanylate binding protein 1, interferon-inducible, 67kDa | -2.1 |
| 221485_at | 9334 | B4GALT5 | UDP-Gal:betaGlcNAc beta 1,4- galactosyltransferase, polypeptide 5 | -2.1 |
| 225726_s_at | 57475 | PLEKHH1 | Pleckstrin homology domain containing, family H (with MyTH4 domain) member 1 | -2.1 |
| 215787_at | 59 | ACTA2 | Actin, alpha 2, smooth muscle, aorta | -2.1 |
| 1558111_at | 4154 | MBNL1 | Muscleblind-like (Drosophila) | -2.1 |
| 204967_at | 357 | SHROOM2 | Shroom family member 2 | -2.1 |
| 204602_at | 22943 | DKK1 | Dickkopf homolog 1 (Xenopus laevis) | -2.1 |
| 231252_at | 151050 | C2ORF67 | Chromosome 2 open reading frame 67 | -2.1 |
| 202068_s_at | 3949 | LDLR | Low density lipoprotein receptor | -2.1 |
| 1555274_a_at | 85465 | EPT1 | Selenoprotein I | -2.1 |
| 228168_at | 518 | ATP5G3 | ATP synthase, H+ transporting, mitochondrial F0 complex, subunit C3 (subunit 9) | -2.1 |
| 242260_at | 9782 | MATR3 | Matrin 3 | -2.1 |
| 201326_at | 908 | CCT6A | Chaperonin containing TCP1, subunit 6A (zeta 1) | -2.1 |
| 222853_at | 23767 | FLRT3 | Fibronectin leucine rich transmembrane protein 3 | -2.0 |
| 223449_at | 57556 | SEMA6A | Sema domain, transmembrane domain (TM), and cytoplasmic domain, (semaphorin) 6A | -2.0 |
| 219740_at | 79805 | VASH2 | Vasohibin 2 | -2.0 |
| 224694_at | 84168 | ANTXR1 | Anthrax toxin receptor 1 | -2.0 |
| 205448_s_at | 7786 | MAP3K12 | Mitogen-activated protein kinase kinase kinase 12 | -2.0 |
| 223394_at | 29950 | SERTAD1 | SERTA domain containing 1 | -2.0 |
| 212944_at | 6526 | SLC5A3 | Solute carrier family 5 (sodium/myo-inositol cotransporter), member 3 | -2.0 |
| 204011_at | 10253 | SPRY2 | Sprouty homolog 2 (Drosophila) | -2.0 |
| 221841_s_at | 9314 | KLF4 | Kruppel-like factor 4 (gut) | -2.0 |
| 212170_at | 10137 | RBM12 | RNA binding motif protein 12; copine I | -2.0 |
| 226109_at | 54149 | C21ORF91 | Chromosome 21 open reading frame 91 | -2.0 |
| 228776_at | 10052 | GJC1 | Gap junction protein, gamma 1, 45kDa | -2.0 |
| 219094_at | 25852 | ARMC8 | Armadillo repeat containing 8 | -2.0 |
| 212658_at | 10184 | LHFPL2 | Lipoma HMGIC fusion partner-like 2 | -2.0 |
| 225978_at | 57494 | RIMKLB | Ribosomal modification protein rimK-like family member B | -2.0 |
| 223079_s_at | 2744 | GLS | Glutaminase | -2.0 |
| 227148_at | 130271 | PLEKHH2 | Pleckstrin homology domain containing, family H (with MyTH4 domain) member 2 | -2.0 |
| 208707_at | 1983 | EIF5 | Eukaryotic translation initiation factor 5 | -2.0 |
| 201370_s_at | 8452 | CUL3 | Cullin 3 | -2.0 |
| 220512_at | 10395 | DLC1 | Deleted in liver cancer 1 | -1.9 |
| 222589_at | 51701 | NLK | Nemo-like kinase | -1.9 |
| 204642_at | 1901 | S1PR1 | Sphingosine-1-phosphate receptor 1 | -1.9 |
| 213387_at | 54454 | ATAD2B | ATPase family, AAA domain containing 2B | -1.9 |
| 203498_at | 10231 | RCAN2 | Regulator of calcineurin 2 | -1.9 |
| 227379_at | 154141 | MBOAT1 | Membrane bound O-acyltransferase domain containing 1 | -1.9 |
| 243711_at | 23576 | DDAH1 | Dimethylarginine dimethylaminohydrolase 1 | -1.9 |
| 206752_s_at | 1677 | DFFB | DNA fragmentation factor, 40kDa, beta polypeptide (caspase-activated DNase) | -1.9 |
| 203692_s_at | 1871 | E2F3 | E2F transcription factor 3 | -1.9 |
| 236535_at | 79677 | SMC6 | Structural maintenance of chromosomes 6 | -1.9 |
| 1569183_a_at | 1121 | CHM | Choroideremia (Rab escort protein 1) | -1.9 |
| 211932_at | 220988 | HNRNPA3 | Heterogeneous nuclear ribonucleoprotein A3 | -1.9 |
| 1556121_at | 4673 | NAP1L1 | Nucleosome assembly protein 1-like 1 | -1.9 |
| 221264_s_at | 23435 | TARDBP | TAR DNA binding protein | -1.9 |
| 212502_at | 84890 | ADO | 2-aminoethanethiol (cysteamine) dioxygenase | -1.9 |
| 203843_at | 6197 | RPS6KA3 | Ribosomal protein S6 kinase, 90kDa, polypeptide 3 | -1.9 |
| 205876_at | 3977 | LIFR | Leukemia inhibitory factor receptor alpha | -1.9 |
| 203908_at | 8671 | SLC4A4 | Solute carrier family 4, sodium bicarbonate cotransporter, member 4 | -1.9 |
| 208900_s_at | 7150 | TOP1 | Topoisomerase (DNA) I | -1.9 |
| 52837_at | 85352 | KIAA1644 | KIAA1644 | -1.9 |
| 203870_at | 64854 | USP46 | Ubiquitin specific peptidase 46 | -1.9 |
| 225539_at | 49854 | ZNF295 | Zinc finger protein 295 | -1.9 |
| 210814_at | 7222 | TRPC3 | Transient receptor potential cation channel, subfamily C, member 3 | -1.9 |
| 203845_at | 8850 | KAT2B | K(lysine) acetyltransferase 2B | -1.9 |
| 225835_at | 6558 | SLC12A2 | Solute carrier family 12 (sodium/potassium/chloride transporters), member 2 | -1.9 |
| 209569_x_at | 27065 | D4S234E | DNA segment on chromosome 4 (unique) 234 expressed sequence | -1.9 |
| 215611_at | 6938 | TCF12 | Transcription factor 12 | -1.8 |
| 205893_at | 22871 | NLGN1 | Neuroligin 1 | -1.8 |
| 226075_at | 80176 | SPSB1 | SplA/ryanodine receptor domain and SOCS box containing 1 | -1.8 |
| 223288_at | 84640 | USP38 | Ubiquitin specific peptidase 38 | -1.8 |
| 207172_s_at | 1009 | CDH11 | Cadherin 11, type 2, OB-cadherin (osteoblast) | -1.8 |
| 226619_at | 29843 | SENP1 | SUMO1/sentrin specific peptidase 1 | -1.8 |
| 227666_at | 166614 | DCLK2 | Doublecortin-like kinase 2 | -1.8 |
| 209068_at | 9987 | HNRPDL | Heterogeneous nuclear ribonucleoprotein D-like | -1.8 |
| 1558356_at | 55075 | UACA | Uveal autoantigen with coiled-coil domains and ankyrin repeats | -1.8 |
| 214814_at | 91746 | YTHDC1 | YTH domain containing 1 | -1.8 |
| 227164_at | 6426 | SFRS1 | Splicing factor, arginine/serine-rich 1 | -1.8 |
| 218330_s_at | 89797 | NAV2 | Neuron navigator 2 | -1.8 |
| 236248_x_at | 93624 | TADA2B | Transcriptional adaptor 2 (ADA2 homolog, yeast)-beta | -1.8 |
| 209348_s_at | 4094 | MAF | V-maf musculoaponeurotic fibrosarcoma oncogene homolog (avian) | -1.8 |
| 225163_at | 55691 | FRMD4A | FERM domain containing 4A | -1.8 |
| 224956_at | 57532 | NUFIP2 | Nuclear fragile X mental retardation protein interacting protein 2 | -1.8 |
| 232067_at | 84553 | C6ORF168 | Chromosome 6 open reading frame 168 | -1.8 |
| 222689_at | 55331 | ACER3 | Alkaline ceramidase 3 | -1.8 |
| 216262_s_at | 60436 | TGIF2 | TGFB-induced factor homeobox 2 | -1.8 |
| 204797_s_at | 2009 | EML1 | Echinoderm microtubule associated protein like 1 | -1.8 |
| 229512_at | 54954 | FAM120C | Family with sequence similarity 120C | -1.8 |
| 231823_s_at | 285590 | SH3PXD2B | SH3 and PX domains 2B | -1.8 |
| 204957_at | 5001 | ORC5L | Origin recognition complex, subunit 5-like (yeast) | -1.8 |
| 1556175_at | 92154 | MTSS1L | Metastasis suppressor 1-like | -1.8 |
| 218864_at | 7145 | TNS1 | Tensin 1 | -1.8 |
| 202470_s_at | 11052 | CPSF6 | Cleavage and polyadenylation specific factor 6, 68kDa | -1.8 |
| 228627_at | 11011 | TLK2 | Tousled-like kinase 2 | -1.8 |
| 229713_at | 5305 | PIP4K2A | Phosphatidylinositol-5-phosphate 4-kinase, type II, alpha | -1.8 |
| 204092_s_at | 6790 | AURKA | Aurora kinase A; aurora kinase A pseudogene 1 | -1.8 |
| 238592_at | 27295 | PDLIM3 | PDZ and LIM domain 3 | -1.8 |
| 217771_at | 51280 | GOLM1 | Golgi membrane protein 1 | -1.8 |
| 222640_at | 1788 | DNMT3A | DNA (cytosine-5-)-methyltransferase 3 alpha | -1.8 |
| 205782_at | 2252 | FGF7 | Fibroblast growth factor 7 (keratinocyte growth factor) | -1.8 |
| 204915_s_at | 6664 | SOX11 | SRY (sex determining region Y)-box 11 | -1.8 |
| 225805_at | 3192 | HNRNPU | Heterogeneous nuclear ribonucleoprotein U (scaffold attachment factor A) | -1.8 |
| 225316_at | 84879 | MFSD2A | Major facilitator superfamily domain containing 2 | -1.8 |
| 232231_at | 860 | RUNX2 | Runt-related transcription factor 2 | -1.8 |
| 218566_s_at | 26973 | CHORDC1 | Cysteine and histidine-rich domain (CHORD)-containing 1 | -1.8 |
| 211347_at | 8555 | CDC14B | CDC14 cell division cycle 14 homolog B (S. cerevisiae) | -1.8 |
| 228490_at | 11057 | ABHD2 | Abhydrolase domain containing 2 | -1.8 |
| 218091_at | 3267 | AGFG1 | ArfGAP with FG repeats 1 | -1.8 |
| 202805_s_at | 4363 | ABCC1 | ATP-binding cassette, sub-family C (CFTR/MRP), member 1 | -1.8 |
| 204935_at | 5771 | PTPN2 | Protein tyrosine phosphatase, non-receptor type 2 | -1.8 |
| 209515_s_at | 5873 | RAB27A | RAB27A, member RAS oncogene family | -1.8 |
| 225368_at | 28996 | HIPK2 | Homeodomain interacting protein kinase 2 | -1.8 |
| 238732_at | 255631 | COL24A1 | Collagen, type XXIV, alpha 1 | -1.8 |
| 203957_at | 1876 | E2F6 | E2F transcription factor 6 | -1.7 |
| 212708_at | 339287 | MSL1 | Male-specific lethal 1 homolog (Drosophila) | -1.7 |
| 235348_at | 84945 | ABHD13 | Abhydrolase domain containing 13 | -1.7 |
| 208930_s_at | 3609 | ILF3 | Interleukin enhancer binding factor 3, 90kDa | -1.7 |
| 202508_s_at | 6616 | SNAP25 | Synaptosomal-associated protein, 25kDa | -1.7 |
| 209681_at | 10560 | SLC19A2 | Solute carrier family 19 (thiamine transporter), member 2 | -1.7 |
| 225070_at | 116150 | NUS1 | Nuclear undecaprenyl pyrophosphate synthase 1 pseudogene | -1.7 |
| 226868_at | 283464 | GXYLT1 | Glycosyltransferase 8 domain containing 3 | -1.7 |
| 237333_at | 5928 | RBBP4 | Hypothetical LOC642954; retinoblastoma binding protein 4 | -1.7 |
| 205330_at | 4330 | MN1 | Meningioma (disrupted in balanced translocation) 1 | -1.7 |
| 224793_s_at | 7046 | TGFBR1 | Transforming growth factor, beta receptor 1 | -1.7 |
| 205256_at | 9880 | ZBTB39 | Zinc finger and BTB domain containing 39 | -1.7 |
| 225954_s_at | 90007 | MIDN | Midnolin | -1.7 |
| 208922_s_at | 10482 | NXF1 | Nuclear RNA export factor 1 | -1.7 |
| 218793_s_at | 6322 | SCML1 | Sex comb on midleg-like 1 (Drosophila) | -1.7 |
| 236313_at | 1030 | CDKN2B | Cyclin-dependent kinase inhibitor 2B (p15, inhibits CDK4) | -1.7 |
| 239143_x_at | 51444 | RNF138 | Ring finger protein 138 | -1.7 |
| 203137_at | 9589 | WTAP | Wilms tumor 1 associated protein | -1.7 |
| 220143_x_at | 55692 | LUC7L | LUC7-like (S. cerevisiae) | -1.7 |
| 239233_at | 55704 | CCDC88A | Coiled-coil domain containing 88A | -1.7 |
| 225888_at | 80018 | NAA25 | Chromosome 12 open reading frame 30 | -1.7 |
| 218829_s_at | 55636 | CHD7 | Chromodomain helicase DNA binding protein 7 | -1.7 |
| 225627_s_at | 57685 | CACHD1 | Cache domain containing 1 | -1.7 |
| 201626_at | 3638 | INSIG1 | Insulin induced gene 1 | -1.7 |
| 221643_s_at | 473 | RERE | Arginine-glutamic acid dipeptide (RE) repeats | -1.7 |
| 204440_at | 9308 | CD83 | CD83 molecule | -1.7 |
| 217904_s_at | 23621 | BACE1 | Beta-site APP-cleaving enzyme 1 | -1.7 |
| 244764_at | 59269 | HIVEP3 | Human immunodeficiency virus type I enhancer binding protein 3 | -1.7 |
| 235068_at | 340481 | ZDHHC21 | Zinc finger, DHHC-type containing 21 | -1.7 |
| 209838_at | 9318 | COPS2 | COP9 constitutive photomorphogenic homolog subunit 2 (Arabidopsis) | -1.7 |
| 219079_at | 51167 | CYB5R4 | Cytochrome b5 reductase 4 | -1.7 |
| 225601_at | 3149 | HMGB3 | Similar to high mobility group box 3; high-mobility group box 3 | -1.7 |
| 218128_at | 4801 | NFYB | Nuclear transcription factor Y, beta | -1.7 |
| 230875_s_at | 23250 | ATP11A | ATPase, class VI, type 11A | -1.7 |
| 213469_at | 80055 | PGAP1 | Post-GPI attachment to proteins 1 | -1.7 |
| 230023_at | 387338 | NSUN4 | NOL1/NOP2/Sun domain family, member 4 | -1.7 |
| 233177_s_at | 25953 | PNKD | Paroxysmal nonkinesigenic dyskinesia | -1.7 |
| 228287_at | 84289 | ING5 | Inhibitor of growth family, member 5 | -1.7 |
| 223442_at | 84276 | NICN1 | Nicolin 1 | -1.7 |
| 226811_at | 54855 | FAM46C | Family with sequence similarity 46, member C | -1.7 |
| 225268_at | 3840 | KPNA4 | Karyopherin alpha 4 (importin alpha 3) | -1.7 |
| 223409_at | 3607 | FOXK2 | Forkhead box K2 | -1.7 |
| 225532_at | 91768 | CABLES1 | Cdk5 and Abl enzyme substrate 1 | -1.7 |
| 228416_at | 92 | ACVR2A | Activin A receptor, type IIA | -1.7 |
| 212751_at | 7334 | UBE2N | Ubiquitin-conjugating enzyme E2N (UBC13 homolog, yeast) | -1.7 |
| 217725_x_at | 26135 | SERBP1 | SERPINE1 mRNA binding protein 1 | -1.6 |
| 218126_at | 55177 | FAM82A2 | Family with sequence similarity 82, member A2 | -1.6 |
| 225688_s_at | 90102 | PHLDB2 | Pleckstrin homology-like domain, family B, member 2 | -1.6 |
| 1557181_s_at | 399947 | C11ORF87 | Chromosome 11 open reading frame 87 | -1.6 |
| 220334_at | 26575 | RGS17 | Regulator of G-protein signaling 17 | -1.6 |
| 209793_at | 2890 | GRIA1 | Glutamate receptor, ionotropic, AMPA 1 | -1.6 |
| 213309_at | 23228 | PLCL2 | Phospholipase C-like 2 | -1.6 |
| 231841_s_at | 57608 | KIAA1462 | KIAA1462 | -1.6 |
| 205321_at | 1968 | EIF2S3 | Eukaryotic translation initiation factor 2, subunit 3 gamma, 52kDa | -1.6 |
| 213390_at | 23211 | ZC3H4 | Zinc finger CCCH-type containing 4 | -1.6 |
| 233809_at | 25764 | SERF2 | Chromosome 15 open reading frame 63; small EDRK-rich factor 2 | -1.6 |
| 222471_s_at | 56888 | KCMF1 | Potassium channel modulatory factor 1 | -1.6 |
| 205251_at | 8864 | PER2 | Period homolog 2 (Drosophila) | -1.6 |
| 212990_at | 8867 | SYNJ1 | Synaptojanin 1 | -1.6 |
| 215188_at | 8428 | STK24 | Serine/threonine kinase 24 (STE20 homolog, yeast) | -1.6 |
| 235791_x_at | 1105 | CHD1 | Chromodomain helicase DNA binding protein 1 | -1.6 |
| 231974_at | 8085 | MLL2 | Myeloid/lymphoid or mixed-lineage leukemia 2 | -1.6 |
| 228138_at | 221785 | ZNF498 | Znc finger protein 498 | -1.6 |
| 211270_x_at | 5725 | PTBP1 | Polypyrimidine tract binding protein 1 | -1.6 |
| 224368_s_at | 57446 | NDRG3 | NDRG family member 3 | -1.6 |
| 225042_s_at | 81566 | CSRNP2 | Cysteine-serine-rich nuclear protein 2 | -1.6 |
| 202543_s_at | 2764 | GMFB | Glia maturation factor, beta | -1.6 |
| 226041_at | 222236 | NAPEPLD | N-acyl phosphatidylethanolamine phospholipase D | -1.6 |
| 208962_s_at | 3992 | FADS1 | Fatty acid desaturase 1 | -1.6 |
| 204357_s_at | 3984 | LIMK1 | LIM domain kinase 1 | -1.6 |
| 213410_at | 26098 | C10ORF137 | Chromosome 10 open reading frame 137 | -1.6 |
| 210802_s_at | 27292 | DIMT1L | DIM1 dimethyladenosine transferase 1-like (S. cerevisiae) | -1.6 |
| 208835_s_at | 51747 | LUC7L3 | Cisplatin resistance-associated overexpressed protein | -1.6 |
| 238693_at | 80012 | PHC3 | Polyhomeotic homolog 3 (Drosophila) | -1.6 |
| 218502_s_at | 7227 | TRPS1 | Trichorhinophalangeal syndrome I | -1.6 |
| 205795_at | 9369 | NRXN3 | Neurexin 3 | -1.6 |
| 212882_at | 23276 | KLHL18 | Kelch-like 18 (Drosophila) | -1.6 |
| 225887_at | 80209 | C13ORF23 | Chromosome 13 open reading frame 23 | -1.6 |
| 1487_at | 2101 | ESRRA | Estrogen-related receptor alpha | -1.6 |
| 212703_at | 83660 | TLN2 | Talin 2 | -1.6 |
| 201957_at | 4660 | PPP1R12B | Protein phosphatase 1, regulatory (inhibitor) subunit 12B | -1.6 |
| 225972_at | 169200 | TMEM64 | Transmembrane protein 64 | -1.6 |
| 223282_at | 10194 | TSHZ1 | Teashirt zinc finger homeobox 1 | -1.6 |
| 209042_s_at | 7327 | UBE2G2 | Ubiquitin-conjugating enzyme E2G 2 (UBC7 homolog, yeast) | -1.6 |
| 226056_at | 57514 | ARHGAP31 | Cdc42 GTPase-activating protein | -1.6 |
| 226029_at | 57216 | VANGL2 | Vang-like 2 (van gogh, Drosophila) | -1.6 |
| 217649_at | 7763 | ZFAND5 | Similar to zinc finger, AN1-type domain 5; zinc finger, AN1-type domain 5 | -1.6 |
| 218896_s_at | 55421 | C17ORF85 | Chromosome 17 open reading frame 85 | -1.6 |
| 228802_at | 348093 | RBPMS2 | RNA binding protein with multiple splicing 2 | -1.6 |
| 233005_at | 54680 | ZNHIT6 | Zinc finger, HIT type 6 | -1.6 |
| 212461_at | 51582 | AZIN1 | Antizyme inhibitor 1 | -1.6 |
| 225459_at | 154810 | AMOTL1 | Angiomotin like 1 | -1.6 |
| 225606_at | 10018 | BCL2L11 | BCL2-like 11 (apoptosis facilitator) | -1.6 |
| 226475_at | 55007 | FAM118A | Family with sequence similarity 118, member A | -1.6 |
| 235024_at | 79960 | PHF17 | PHD finger protein 17 | -1.6 |
| 1553218_a_at | 84450 | ZNF512 | Zinc finger protein 512 | -1.6 |
| 227239_at | 84668 | FAM126A | Family with sequence similarity 126, member A | -1.6 |
| 229134_at | 81839 | VANGL1 | Vang-like 1 (van gogh, Drosophila) | -1.6 |
| 231975_s_at | 166968 | MIER3 | Mesoderm induction early response 1, family member 3 | -1.6 |
| 223213_s_at | 11244 | ZHX1 | Zinc fingers and homeoboxes 1 | -1.6 |
| 235521_at | 3200 | HOXA3 | Homeobox A3 | -1.6 |
| 226367_at | 5927 | KDM5A | Lysine (K)-specific demethylase 5A | -1.6 |
| 201811_x_at | 9467 | SH3BP5 | SH3-domain binding protein 5 (BTK-associated) | -1.6 |
| 219800_s_at | 79896 | THNSL1 | Threonine synthase-like 1 (S. cerevisiae) | -1.6 |
| 203706_s_at | 8324 | FZD7 | Frizzled homolog 7 (Drosophila) | -1.6 |
| 227542_at | 9306 | SOCS6 | Suppressor of cytokine signaling 6 | -1.6 |
| 1554178_a_at | 285172 | FAM126B | Family with sequence similarity 126, member B | -1.6 |
| 204742_s_at | 23047 | PDS5B | PDS5, regulator of cohesion maintenance, homolog B (S. cerevisiae) | -1.6 |
| 219073_s_at | 114884 | OSBPL10 | Oxysterol binding protein-like 10 | -1.6 |
| 227978_s_at | 284273 | ZADH2 | Zinc binding alcohol dehydrogenase domain containing 2 | -1.6 |
| 201934_at | 80335 | WDR82 | WD repeat domain 82 | -1.6 |
| 213552_at | 26035 | GLCE | Glucuronic acid epimerase | -1.5 |
| 200690_at | 3313 | HSPA9 | Heat shock 70kDa protein 9 (mortalin) | -1.5 |
| 227314_at | 3673 | ITGA2 | Integrin, alpha 2 (CD49B, alpha 2 subunit of VLA-2 receptor) | -1.5 |
| 212655_at | 23174 | ZCCHC14 | Zinc finger, CCHC domain containing 14 | -1.5 |
| 231907_at | 27 | ABL2 | V-abl Abelson murine leukemia viral oncogene homolog 2 (arg, Abelson-related gene) | -1.5 |
| 218031_s_at | 1112 | FOXN3 | Forkhead box N3 | -1.5 |
| 217779_s_at | 55629 | PNRC2 | Proline-rich nuclear receptor coactivator 2 | -1.5 |
| 223298_s_at | 51251 | NT5C3 | 5'-nucleotidase, cytosolic III | -1.5 |
| 212728_at | 1741 | DLG3 | Discs, large homolog 3 (Drosophila) | -1.5 |
| 219682_s_at | 6926 | TBX3 | T-box 3 | -1.5 |
| 1554053_at | 10558 | SPTLC1 | Serine palmitoyltransferase, long chain base subunit 1 | -1.5 |
| 242019_at | 253782 | LASS6 | LAG1 homolog, ceramide synthase 6 | -1.5 |
| 205525_at | 800 | CALD1 | Caldesmon 1 | -1.5 |
| 212322_at | 8879 | SGPL1 | Sphingosine-1-phosphate lyase 1 | -1.5 |
| 206091_at | 4148 | MATN3 | Matrilin 3 | -1.5 |
| 208939_at | 22929 | SEPHS1 | Selenophosphate synthetase 1 | -1.5 |
| 222526_at | 54815 | GATAD2A | GATA zinc finger domain containing 2A | -1.5 |
| 221234_s_at | 60468 | BACH2 | BTB and CNC homology 1, basic leucine zipper transcription factor 2 | -1.5 |
| 1556116_s_at | 3842 | TNPO1 | Transportin 1 | -1.5 |
| 212666_at | 57154 | SMURF1 | SMAD specific E3 ubiquitin protein ligase 1 | -1.5 |
| 225556_at | 203547 | VMA21 | VMA21 vacuolar H+-ATPase homolog (S. cerevisiae) | -1.5 |
| 225179_at | 3093 | UBE2K | Ubiquitin-conjugating enzyme E2K (UBC1 homolog, yeast) | -1.5 |
| 205773_at | 22849 | CPEB3 | Cytoplasmic polyadenylation element binding protein 3 | -1.5 |
| 209981_at | 27254 | CSDC2 | Cold shock domain containing C2, RNA binding | -1.5 |
| 239297_at | 57604 | C8ORF79 | Chromosome 8 open reading frame 79 | -1.5 |
| 235043_at | 116224 | FAM122A | Family with sequence similarity 122A | -1.5 |
| 226363_at | 10057 | ABCC5 | ATP-binding cassette, sub-family C (CFTR/MRP), member 5 | -1.5 |
| 224835_at | 56261 | GPCPD1 | Hypothetical protein KIAA1434 | -1.5 |
| 227436_at | 56957 | OTUD7B | OTU domain containing 7B | -1.5 |
| 208912_s_at | 1267 | CNP | 2',3'-cyclic nucleotide 3' phosphodiesterase | -1.5 |
| 205463_s_at | 5154 | PDGFA | Platelet-derived growth factor alpha polypeptide | -1.5 |
| 218265_at | 79048 | SECISBP2 | SECIS binding protein 2 | -1.5 |
| 207332_s_at | 7037 | TFRC | Transferrin receptor (p90, CD71) | -1.5 |
| 216952_s_at | 84823 | LMNB2 | Lamin B2 | -1.5 |
| 216248_s_at | 4929 | NR4A2 | Nuclear receptor subfamily 4, group A, member 2 | -1.5 |
| 235440_at | 144108 | SPTY2D1 | SPT2, Suppressor of Ty, domain containing 1 (S. cerevisiae) | -1.5 |
| 214230_at | 998 | CDC42P2 | Cell division cycle 42 (GTP binding protein, 25kDa) | -1.5 |
| 235486_at | 25758 | C11ORF41 | Chromosome 11 open reading frame 41 | -1.5 |
| 210002_at | 2627 | GATA6 | GATA binding protein 6 | -1.5 |
| 222393_s_at | 80218 | NAA50 | N-acetyltransferase 13 (GCN5-related) | -1.5 |
| 57739_at | 373863 | DND1 | Dead end homolog 1 (zebrafish); similar to dead end homolog 1 | -1.5 |
| 209190_s_at | 1729 | DIAPH1 | Diaphanous homolog 1 (Drosophila) | -1.5 |
| 244660_at | 1994 | ELAVL1 | ELAV (embryonic lethal, abnormal vision, Drosophila)-like 1 (Hu antigen R) | -1.5 |
| 202860_at | 9909 | DENND4B | DENN/MADD domain containing 4B | -1.5 |
| 203765_at | 25801 | GCA | Grancalcin, EF-hand calcium binding protein | -1.5 |
| 203128_at | 9517 | SPTLC2 | Serine palmitoyltransferase, long chain base subunit 2 | -1.5 |
| 1555982_at | 9765 | ZFYVE16 | Zinc finger, FYVE domain containing 16 | -1.5 |
| 201376_s_at | 3185 | HNRNPF | Heterogeneous nuclear ribonucleoprotein F | -1.5 |
| 228445_at | 84883 | AIFM2 | Apoptosis-inducing factor, mitochondrion-associated, 2 | -1.5 |
| 209320_at | 109 | ADCY3 | Adenylate cyclase 3 | -1.5 |
| 225831_at | 7798 | LUZP1 | Leucine zipper protein 1 | -1.5 |
| 224929_at | 340061 | TMEM173 | Transmembrane protein 173 | -1.5 |
| 204435_at | 9818 | NUPL1 | Nucleoporin like 1 | -1.5 |
| 237159_x_at | 130340 | AP1S3 | Adaptor-related protein complex 1, sigma 3 subunit | -1.5 |
| 201739_at | 6446 | SGK1 | Serum/glucocorticoid regulated kinase 1 | -1.5 |
| 222999_s_at | 81669 | CCR6 | Cyclin L2; chemokine (C-C motif) receptor 6 | -1.5 |
| 226720_at | 114825 | PWWP2A | PWWP domain containing 2A | -1.5 |
| 225659_at | 339745 | SPOPL | Speckle-type POZ protein-like | -1.5 |
| 202272_s_at | 23219 | FBXO28 | F-box protein 28 | -1.5 |
| 228846_at | 4084 | MXD1 | MAX dimerization protein 1 | -1.5 |
| 213029_at | 4781 | NFIB | Nuclear factor I/B | -1.5 |
| 203743_s_at | 6996 | TDG | Thymine-DNA glycosylase | -1.5 |
| 225633_at | 147991 | DPY19L3 | Dpy-19-like 3 (C. elegans) | -1.5 |
| 225144_at | 659 | BMPR2 | Bone morphogenetic protein receptor, type II (serine/threonine kinase) | -1.4 |
| 224817_at | 9644 | SH3PXD2A | SH3 and PX domains 2A | -1.4 |
| 228443_s_at | 387893 | SETD8 | SET domain containing (lysine methyltransferase) 8 | -1.4 |
| 1553749_at | 143684 | FAM76B | Family with sequence similarity 76, member B | -1.4 |
| 235615_at | 5229 | PGGT1B | Protein geranylgeranyltransferase type I, beta subunit | -1.4 |
| 205215_at | 6045 | RNF2 | Ring finger protein 2 | -1.4 |
| 203414_at | 23531 | MMD | Monocyte to macrophage differentiation-associated | -1.4 |

* Fold change calculated by SAM analysis of microarrays from young and senescent IMR90 fibroblasts.
