# Supplementary material for: Deep Sequencing Reveals Novel MicroRNAs and Regulation of MicroRNA Expression during Cell Senescence
Source: PLoS One. 2011 May 26;6(5):e20509. doi: 10.1371/journal.pone.0020509 (PMC3102725; doi:10.1371/journal.pone.0020509)
Supplement: Table S4 — Potential target genes upregulated by senescence-induced miRNA underexpression in IMR90 fibroblasts. (DOC) [file pone.0020509.s005.doc]

**Table S4**. Potential target genes upregulated by senescence-induced miRNA underexpression in IMR90 fibroblasts.

| **Affy id** | **Entrez gene id** | **Gene symbol** | **Gene name** | **Fold change*** |
| --- | --- | --- | --- | --- |
| 225342_at | 205 | AK4 | Adenylate kinase 3-like 2; adenylate kinase 3-like 1 | 8.0 |
| 218149_s_at | 55893 | ZNF395 | Zinc finger protein 395 | 7.2 |
| 233882_s_at | 80031 | SEMA6D | Sema domain, transmembrane domain (TM), and cytoplasmic domain, (semaphorin) 6D | 5.6 |
| 202973_x_at | 10144 | FAM13A | Family with sequence similarity 13, member A | 4.3 |
| 213845_at | 2898 | GRIK2 | Glutamate receptor, ionotropic, kainate 2 | 3.9 |
| 204298_s_at | 4015 | LOX | Lysyl oxidase | 3.7 |
| 228153_at | 255488 | RNF144B | Ring finger protein 144B | 3.4 |
| 204491_at | 5144 | PDE4D | Phosphodiesterase 4D, cAMP-specific (phosphodiesterase E3 dunce homolog, Drosophila) | 3.1 |
| 223062_s_at | 29968 | PSAT1 | Chromosome 8 open reading frame 62; phosphoserine aminotransferase 1 | 3.0 |
| 201295_s_at | 26118 | WSB1 | WD repeat and SOCS box-containing 1 | 3.0 |
| 224952_at | 26115 | TANC2 | Tetratricopeptide repeat, ankyrin repeat and coiled-coil containing 2 | 2.9 |
| 203278_s_at | 51317 | PHF21A | PHD finger protein 21A | 2.9 |
| 203556_at | 22882 | ZHX2 | Zinc fingers and homeoboxes 2 | 2.8 |
| 227649_s_at | 23380 | SRGAP2 | SLIT-ROBO Rho GTPase activating protein 2 | 2.7 |
| 200737_at | 5230 | PGK1 | Phosphoglycerate kinase 1 | 2.7 |
| 203373_at | 8835 | SOCS2 | Suppressor of cytokine signaling 2 | 2.7 |
| 215177_s_at | 3655 | ITGA6 | Integrin, alpha 6 | 2.6 |
| 203910_at | 9411 | ARHGAP29 | Rho GTPase activating protein 29 | 2.6 |
| 222593_s_at | 65244 | SPATS2 | Spermatogenesis associated, serine-rich 2 | 2.5 |
| 203543_s_at | 687 | KLF9 | Kruppel-like factor 9 | 2.5 |
| 200815_s_at | 5048 | PAFAH1B1 | Platelet-activating factor acetylhydrolase, isoform Ib, subunit 1 (45kDa) | 2.5 |
| 209737_at | 9863 | MAGI2 | Membrane associated guanylate kinase, WW and PDZ domain containing 2 | 2.4 |
| 226352_at | 133746 | JMY | Junction mediating and regulatory protein, p53 cofactor | 2.4 |
| 209318_x_at | 5325 | PLAGL1 | Pleiomorphic adenoma gene-like 1 | 2.4 |
| 239202_at | 5865 | RAB3B | RAB3B, member RAS oncogene family | 2.4 |
| 1552712_a_at | 23057 | NMNAT2 | Nicotinamide nucleotide adenylyltransferase 2 | 2.3 |
| 219937_at | 29953 | TRHDE | Thyrotropin-releasing hormone degrading enzyme | 2.3 |
| 212445_s_at | 23327 | NEDD4L | Neural precursor cell expressed, developmentally down-regulated 4-like | 2.3 |
| 202730_s_at | 27250 | PDCD4 | Programmed cell death 4 (neoplastic transformation inhibitor) | 2.3 |
| 201917_s_at | 55186 | SLC25A36 | Solute carrier family 25, member 36 | 2.3 |
| 214452_at | 586 | BCAT1 | Branched chain aminotransferase 1, cytosolic | 2.3 |
| 225717_at | 80856 | KIAA1715 | KIAA1715 | 2.3 |
| 1558208_at | 23435 | TARDBP | TAR DNA binding protein | 2.3 |
| 224851_at | 1021 | CDK6 | Cyclin-dependent kinase 6 | 2.2 |
| 241789_at | 27303 | RBMS3 | RNA binding motif, single stranded interacting protein | 2.2 |
| 205923_at | 5649 | RELN | Reelin | 2.2 |
| 213906_at | 4603 | MYBL1 | V-myb myeloblastosis viral oncogene homolog (avian)-like 1 | 2.2 |
| 225026_at | 84181 | CHD6 | Chromodomain helicase DNA binding protein 6 | 2.2 |
| 209357_at | 10370 | CITED2 | Cbp/p300-interacting transactivator, with Glu/Asp-rich carboxy-terminal domain, 2 | 2.2 |
| 201534_s_at | 5412 | UBL3 | Ubiquitin-like 3 | 2.1 |
| 228777_at | 143879 | KBTBD3 | Kelch repeat and BTB (POZ) domain containing 3 | 2.1 |
| 222549_at | 9076 | CLDN1 | Claudin 1 | 2.1 |
| 204597_x_at | 6781 | STC1 | Stanniocalcin 1 | 2.1 |
| 204527_at | 4644 | MYO5A | Myosin VA (heavy chain 12, myoxin) | 2.1 |
| 227713_at | 84056 | KATNAL1 | Katanin p60 subunit A-like 1 | 2.0 |
| 207543_s_at | 5033 | P4HA1 | Prolyl 4-hydroxylase, alpha polypeptide I | 2.0 |
| 205372_at | 5324 | PLAG1 | Pleiomorphic adenoma gene 1 | 2.0 |
| 201108_s_at | 7057 | THBS1 | Thrombospondin 1 | 2.0 |
| 209102_s_at | 26959 | HBP1 | HMG-box transcription factor 1 | 2.0 |
| 222730_s_at | 51201 | ZDHHC2 | Zinc finger, DHHC-type containing 2 | 2.0 |
| 212851_at | 23142 | DCUN1D4 | DCN1, defective in cullin neddylation 1, domain containing 4 (S. cerevisiae) | 2.0 |
| 205105_at | 4124 | MAN2A1 | Mannosidase, alpha, class 2A, member 1 | 2.0 |
| 202336_s_at | 5066 | PAM | Peptidylglycine alpha-amidating monooxygenase | 2.0 |
| 230109_at | 27115 | PDE7B | Phosphodiesterase 7B | 2.0 |
| 1559400_s_at | 5069 | PAPPA | PAPPA antisense RNA (non-protein coding); pregnancy-associated plasma protein A, pappalysin 1 | 2.0 |
| 1558116_x_at | 8434 | RECK | Reversion-inducing-cysteine-rich protein with kazal motifs | 2.0 |
| 225956_at | 153222 | C5orf41 | Chromosome 5 open reading frame 41 | 2.0 |
| 205005_s_at | 9397 | NMT2 | N-myristoyltransferase 2 | 2.0 |
| 202499_s_at | 6515 | SLC2A3 | Solute carrier family 2 (facilitated glucose transporter), member 3 | 1.9 |
| 235635_at | 394 | ARHGAP5 | Rho GTPase activating protein 5 | 1.9 |
| 200921_s_at | 694 | BTG1 | B-cell translocation gene 1, anti-proliferative | 1.9 |
| 204710_s_at | 26100 | WIPI2 | WD repeat domain, phosphoinositide interacting 2 | 1.9 |
| 227295_at | 121457 | IKBIP | IKK interacting protein | 1.9 |
| 206591_at | 5896 | RAG1 | Recombination activating gene 1 | 1.9 |
| 213792_s_at | 3643 | INSR | Insulin receptor | 1.9 |
| 217433_at | 6867 | TACC1 | Transforming, acidic coiled-coil containing protein 1 | 1.9 |
| 227809_at | 376940 | ZC3H6 | Zinc finger CCCH-type containing 6 | 1.9 |
| 1561079_at | 23243 | ANKRD28 | Ankyrin repeat domain 28 | 1.9 |
| 218268_at | 64786 | TBC1D15 | TBC1 domain family, member 15 | 1.9 |
| 200758_s_at | 4779 | NFE2L1 | Nuclear factor (erythroid-derived 2)-like 1 | 1.9 |
| 221962_s_at | 7328 | UBE2H | Ubiquitin-conjugating enzyme E2H (UBC8 homolog, yeast) | 1.9 |
| 218277_s_at | 79665 | DHX40 | Similar to DEAH (Asp-Glu-Ala-His) box polypeptide 40; DEAH (Asp-Glu-Ala-His) box polypeptide 40 | 1.9 |
| 218603_at | 51696 | HECA | Headcase homolog (Drosophila) | 1.9 |
| 204569_at | 22858 | ICK | Intestinal cell (MAK-like) kinase | 1.9 |
| 225327_at | 56204 | KIAA1370 | KIAA1370 | 1.9 |
| 214688_at | 7091 | TLE4 | Transducin-like enhancer of split 4 (E(sp1) homolog, Drosophila) | 1.8 |
| 229256_at | 283209 | PGM2L1 | Phosphoglucomutase 2-like 1 | 1.8 |
| 225263_at | 9394 | HS6ST1 | Heparan sulfate 6-O-sulfotransferase 1 | 1.8 |
| 231251_at | 147179 | WIPF2 | WAS/WASL interacting protein family, member 2 | 1.8 |
| 224678_at | 57148 | RALGAPB | KIAA1219 | 1.8 |
| 225912_at | 94241 | TP53INP1 | Tumor protein p53 inducible nuclear protein 1 | 1.8 |
| 219771_at | 54885 | TBC1D8B | TBC1 domain family, member 8B (with GRAM domain) | 1.8 |
| 209921_at | 23657 | SLC7A11 | Solute carrier family 7, (cationic amino acid transporter, y+ system) member 11 | 1.8 |
| 211075_s_at | 961 | CD47 | CD47 molecule | 1.8 |
| 201195_s_at | 8140 | SLC7A5 | Solute carrier family 7 (cationic amino acid transporter, y+ system), member 5 | 1.8 |
| 227179_at | 27067 | STAU2 | Staufen, RNA binding protein, homolog 2 (Drosophila) | 1.8 |
| 205882_x_at | 120 | ADD3 | Adducin 3 (gamma) | 1.8 |
| 241384_x_at | 256356 | GK5 | Glycerol kinase 5 (putative) | 1.8 |
| 212914_at | 23492 | CBX7 | Chromobox homolog 7 | 1.8 |
| 201000_at | 16 | AARS | Alanyl-tRNA synthetase | 1.8 |
| 214436_at | 25827 | FBXL2 | F-box and leucine-rich repeat protein 2 | 1.8 |
| 202704_at | 10140 | TOB1 | Transducer of ERBB2, 1 | 1.8 |
| 228149_at | 154743 | C7orf60 | Chromosome 7 open reading frame 60 | 1.8 |
| 225051_at | 2035 | EPB41 | Erythrocyte membrane protein band 4.1 (elliptocytosis 1, RH-linked) | 1.8 |
| 204206_at | 4335 | MNT | MAX binding protein | 1.8 |
| 228220_at | 115548 | FCHO2 | FCH domain only 2 | 1.8 |
| 231930_at | 55531 | ELMOD1 | ELMO/CED-12 domain containing 1 | 1.8 |
| 1558682_at | 8091 | HMGA2 | High mobility group AT-hook 2 | 1.8 |
| 201236_s_at | 7832 | BTG2 | BTG family, member 2 | 1.8 |
| 220800_s_at | 29766 | TMOD3 | Tropomodulin 3 (ubiquitous) | 1.8 |
| 212076_at | 4297 | MLL | Myeloid/lymphoid or mixed-lineage leukemia | 1.7 |
| 202739_s_at | 5257 | PHKB | Phosphorylase kinase, beta | 1.7 |
| 225295_at | 57181 | SLC39A10 | Solute carrier family 39 (zinc transporter), member 10 | 1.7 |
| 223430_at | 23235 | SIK2 | Salt-inducible kinase 2 | 1.7 |
| 225330_at | 3480 | IGF1R | Insulin-like growth factor 1 receptor | 1.7 |
| 203042_at | 3920 | LAMP2 | Lysosomal-associated membrane protein 2 | 1.7 |
| 204917_s_at | 4300 | MLLT3 | Myeloid/lymphoid or mixed-lineage leukemia; translocated to, 3 | 1.7 |
| 223492_s_at | 9208 | LRRFIP1 | Leucine rich repeat (in FLII) interacting protein 1 | 1.7 |
| 225262_at | 2355 | FOSL2 | FOS-like antigen 2 | 1.7 |
| 228248_at | 253260 | RICTOR | RPTOR independent companion of MTOR, complex 2 | 1.7 |
| 203390_s_at | 3797 | KIF3C | Kinesin family member 3C | 1.7 |
| 201578_at | 5420 | PODXL | Podocalyxin-like | 1.7 |
| 239290_at | 9758 | FRMPD4 | FERM and PDZ domain containing 4 | 1.7 |
| 40446_at | 5252 | PHF1 | PHD finger protein 1 | 1.7 |
| 222602_at | 55236 | UBA6 | Ubiquitin-like modifier activating enzyme 6 | 1.7 |
| 202213_s_at | 8450 | CUL4B | Cullin 4B | 1.7 |
| 202627_s_at | 5054 | SERPINE1 | Serpin peptidase inhibitor, clade E (nexin, plasminogen activator inhibitor type 1), member 1 | 1.7 |
| 221036_s_at | 83464 | APH1B | Anterior pharynx defective 1 homolog B (C. elegans) | 1.7 |
| 202778_s_at | 7750 | ZMYM2 | Zinc finger, MYM-type 2 | 1.7 |
| 201655_s_at | 3339 | HSPG2 | Heparan sulfate proteoglycan 2 | 1.7 |
| 222745_s_at | 79768 | C15orf29 | Chromosome 15 open reading frame 29 | 1.7 |
| 205417_s_at | 1605 | DAG1 | Dystroglycan 1 (dystrophin-associated glycoprotein 1) | 1.6 |
| 216550_x_at | 23253 | ANKRD12 | Ankyrin repeat domain 12 | 1.6 |
| 202832_at | 9648 | GCC2 | GRIP and coiled-coil domain containing 2 | 1.6 |
| 202079_s_at | 22906 | TRAK1 | Trafficking protein, kinesin binding 1 | 1.6 |
| 232898_at | 1601 | DAB2 | Disabled homolog 2, mitogen-responsive phosphoprotein (Drosophila) | 1.6 |
| 201637_s_at | 8087 | FXR1 | Fragile X mental retardation, autosomal homolog 1 | 1.6 |
| 203323_at | 858 | CAV2 | Caveolin 2 | 1.6 |
| 226583_at | 400073 | C12orf76 | Chromosome 12 open reading frame 76 | 1.6 |
| 228551_at | 160518 | DENND5B | DENN/MADD domain containing 5B | 1.6 |
| 202641_at | 403 | ARL3 | ADP-ribosylation factor-like 3 | 1.6 |

* Fold change calculated by SAM analysis of microarrays from young and senescent IMR90 fibroblasts.
