# Supplementary material for: Deep Sequencing Reveals Novel MicroRNAs and Regulation of MicroRNA Expression during Cell Senescence
Source: PLoS One. 2011 May 26;6(5):e20509. doi: 10.1371/journal.pone.0020509 (PMC3102725; doi:10.1371/journal.pone.0020509)
Supplement: Table S5 — Potential target genes downregulated by senescence-induced miRNA overexpression in both types of fibroblasts, IMR90 and MRC5. (DOC) [file pone.0020509.s006.doc]

**Table S5**. Potential target genes downregulated by senescence-induced miRNA overexpression in both types of fibroblasts, IMR90 and MRC5.

|  | | | **IMR90 fibroblast (Affymetrix)** | | **MRC5 fibroblast (two-color microarrays)** | |
| --- | --- | --- | --- | --- | --- | --- |
| **Entrez gene id** | **Gene symbol** | **Gene name** | **Probe id** | **FC^1^** | **Probe id** | **FC^2^** |
| 59 | ACTA2 | actin, alpha 2, smooth muscle, aorta | 215787_at | -2.1 | H200014719 | -2.8 |
| 92 | ACVR2A | activin A receptor, type IIA | 228416_at | -1.7 | H200003587 | -1.3 |
| 473 | RERE | arginine-glutamic acid dipeptide (RE) repeats | 221643_s_at | -1.7 | H200014620 | -1.5 |
| 490 | ATP2B1 | ATPase, Ca++ transporting, plasma membrane 1 | 212930_at | -2.1 | H200002948 | -2.1 |
| 860 | RUNX2 | runt-related transcription factor 2 | 232231_at | -1.8 | H200004968 | -1.4 |
| 908 | CCT6A | chaperonin containing TCP1, subunit 6A (zeta 1) | 201326_at | -2.1 | H200006985 | -1.3 |
| 1105 | CHD1 | chromodomain helicase DNA binding protein 1 | 235791_x_at | -1.6 | H200003180 | -1.4 |
| 1112 | FOXN3 | forkhead box N3 | 218031_s_at | -1.5 | H200005405 | -1.6 |
| 1741 | DLG3 | discs, large homolog 3 (Drosophila) | 212728_at | -1.5 | H200001969 | -1.3 |
| 1788 | DNMT3A | DNA (cytosine-5-)-methyltransferase 3 alpha | 222640_at | -1.8 | H200015773 | -1.2 |
| 1983 | EIF5 | eukaryotic translation initiation factor 5 | 208707_at | -2.0 | H200019688 | -1.3 |
| 1994 | ELAVL1 | ELAV (embryonic lethal, abnormal vision, Drosophila)-like 1 (Hu antigen R) | 244660_at | -1.5 | H200002121 | -1.2 |
| 2006 | ELN | elastin | 212670_at | -4.3 | H200001762 | -2.2 |
| 2201 | FBN2 | fibrillin 2 | 203184_at | -4.0 | H200006699 | -1.8 |
| 2252 | FGF7 | fibroblast growth factor 7 (keratinocyte growth factor) | 205782_at | -1.8 | H200007600 | -1.8 |
| 2627 | GATA6 | GATA binding protein 6 | 210002_at | -1.5 | H200005018 | -1.7 |
| 2633 | GBP1 | guanylate binding protein 1, interferon-inducible, 67kDa | 231577_s_at | -2.1 | H200005495 | -1.4 |
| 2882 | GPX7 | glutathione peroxidase 7 | 213170_at | -2.1 | H200004646 | -1.4 |
| 3093 | UBE2K | ubiquitin-conjugating enzyme E2K (UBC1 homolog, yeast) | 225179_at | -1.5 | H200001500 | -1.3 |
| 3149 | HMGB3 | similar to high mobility group box 3; high-mobility group box 3 | 225601_at | -1.7 | H200005496 | -1.5 |
| 3181 | HNRNPA2B1 | heterogeneous nuclear ribonucleoprotein A2/B1 | 225932_s_at | -2.2 | H200015459 | -1.4 |
| 3192 | HNRNPU | heterogeneous nuclear ribonucleoprotein U (scaffold attachment factor A) | 225805_at | -1.8 | H200011258 | -1.3 |
| 3554 | IL1R1 | interleukin 1 receptor, type I | 202948_at | -3.4 | H200006888 | -3.0 |
| 3609 | ILF3 | interleukin enhancer binding factor 3, 90kDa | 208930_s_at | -1.7 | H200016412 | -1.3 |
| 4254 | KITLG | KIT ligand | 226534_at | -3.4 | H200000274 | -2.0 |
| 4363 | ABCC1 | ATP-binding cassette, sub-family C (CFTR/MRP), member 1 | 202805_s_at | -1.8 | H200010321 | -1.3 |
| 4628 | MYH10 | myosin, heavy chain 10, non-muscle | 213067_at | -2.6 | H200009821 | -2.5 |
| 4673 | NAP1L1 | nucleosome assembly protein 1-like 1 | 1556121_at | -1.9 | H200018129 | -1.7 |
| 4781 | NFIB | nuclear factor I/B | 213029_at | -1.5 | H200004230 | -1.4 |
| 4801 | NFYB | nuclear transcription factor Y, beta | 218128_at | -1.7 | H200007126 | -1.7 |
| 4929 | NR4A2 | nuclear receptor subfamily 4, group A, member 2 | 216248_s_at | -1.5 | H200006891 | -2.1 |
| 5156 | PDGFRA | platelet-derived growth factor receptor, alpha polypeptide | 215305_at | -2.5 | H200005974 | -2.6 |
| 5577 | PRKAR2B | protein kinase, cAMP-dependent, regulatory, type II, beta | 203680_at | -5.6 | H200006397 | -1.4 |
| 5725 | PTBP1 | polypyrimidine tract binding protein 1 | 211270_x_at | -1.6 | H200008050 | -1.5 |
| 5771 | PTPN2 | protein tyrosine phosphatase, non-receptor type 2 | 204935_at | -1.8 | H200006978 | -1.5 |
| 5814 | PURB | purine-rich element binding protein B | 235711_at | -2.8 | H200015439 | -1.4 |
| 5873 | RAB27A | RAB27A, member RAS oncogene family | 209515_s_at | -1.8 | H200004987 | -1.7 |
| 5928 | RBBP4 | hypothetical LOC642954; retinoblastoma binding protein 4 | 237333_at | -1.7 | H200002525 | -1.6 |
| 6421 | SFPQ | splicing factor proline/glutamine-rich (polypyrimidine tract binding protein associated) | 201586_s_at | -2.7 | H200008448 | -1.5 |
| 6426 | SFRS1 | splicing factor, arginine/serine-rich 1 | 227164_at | -1.8 | H200014036 | -1.6 |
| 6444 | SGCD | sarcoglycan, delta (35kDa dystrophin-associated glycoprotein) | 213543_at | -2.9 | H200001903 | -2.0 |
| 6446 | SGK1 | serum/glucocorticoid regulated kinase 1 | 201739_at | -1.5 | H200009671 | -2.2 |
| 6616 | SNAP25 | synaptosomal-associated protein, 25kDa | 202508_s_at | -1.7 | H200007108 | -1.6 |
| 6664 | SOX11 | SRY (sex determining region Y)-box 11 | 204915_s_at | -1.8 | H200004198 | -2.2 |
| 6760 | SS18 | synovial sarcoma translocation, chromosome 18 | 202817_s_at | -3.2 | H200013860 | -1.2 |
| 6790 | AURKA | aurora kinase A; aurora kinase A pseudogene 1 | 204092_s_at | -1.8 | H200016261 | -1.9 |
| 6925 | TCF4 | transcription factor 4 | 212385_at | -2.8 | H200009471 | -2.1 |
| 6926 | TBX3 | T-box 3 | 219682_s_at | -1.5 | H200016590 | -1.6 |
| 6938 | TCF12 | transcription factor 12 | 215611_at | -1.8 | H200003059 | -1.7 |
| 6996 | TDG | thymine-DNA glycosylase | 203743_s_at | -1.5 | H200008170 | -1.4 |
| 7046 | TGFBR1 | transforming growth factor, beta receptor 1 | 224793_s_at | -1.7 | H200003807 | -3.3 |
| 7112 | TMPO | thymopoietin | 203432_at | -2.7 | H200001992 | -2.2 |
| 7222 | TRPC3 | transient receptor potential cation channel, subfamily C, member 3 | 210814_at | -1.9 | H200013735 | -2.4 |
| 7334 | UBE2N | ubiquitin-conjugating enzyme E2N (UBC13 homolog, yeast) | 212751_at | -1.7 | H200006084 | -1.3 |
| 7552 | ZNF711 | zinc finger protein 711 | 228988_at | -3.0 | H200019272 | -1.9 |
| 8324 | FZD7 | frizzled homolog 7 (Drosophila) | 203706_s_at | -1.6 | H200008173 | -1.3 |
| 8428 | STK24 | serine/threonine kinase 24 (STE20 homolog, yeast) | 215188_at | -1.6 | H200007776 | -1.3 |
| 8445 | DYRK2 | dual-specificity tyrosine-(Y)-phosphorylation regulated kinase 2 | 202969_at | -2.6 | H200008116 | -1.3 |
| 8555 | CDC14B | CDC14 cell division cycle 14 homolog B (S. cerevisiae) | 211347_at | -1.8 | H200018511 | -1.2 |
| 8613 | PPAP2B | phosphatidic acid phosphatase type 2B | 212230_at | -3.2 | H200008163 | -2.6 |
| 8879 | SGPL1 | sphingosine-1-phosphate lyase 1 | 212322_at | -1.5 | H200014356 | -1.4 |
| 9057 | SLC7A6 | solute carrier family 7 (cationic amino acid transporter, y+ system), member 6 | 203579_s_at | -2.7 | H200001901 | -1.4 |
| 9334 | B4GALT5 | UDP-Gal:betaGlcNAc beta 1,4- galactosyltransferase, polypeptide 5 | 221485_at | -2.1 | H200011525 | -1.4 |
| 9532 | BAG2 | BCL2-associated athanogene 2 | 209406_at | -2.1 | H200005162 | -1.4 |
| 9750 | FAM65B | family with sequence similarity 65, member B | 209829_at | -2.8 | H200011118 | -3.8 |
| 9759 | HDAC4 | histone deacetylase 4 | 204225_at | -2.2 | H200010488 | -1.6 |
| 9765 | ZFYVE16 | zinc finger, FYVE domain containing 16 | 1555982_at | -1.5 | H200007063 | -1.5 |
| 9782 | MATR3 | matrin 3 | 242260_at | -2.1 | H200006559 | -1.3 |
| 9818 | NUPL1 | nucleoporin like 1 | 204435_at | -1.5 | H200010433 | -1.6 |
| 10052 | GJC1 | gap junction protein, gamma 1, 45kDa | 228776_at | -2.0 | H200004647 | -1.5 |
| 10137 | RBM12 | RNA binding motif protein 12; copine I | 212170_at | -2.0 | H200008479 | -1.3 |
| 10184 | LHFPL2 | lipoma HMGIC fusion partner-like 2 | 212658_at | -2.0 | H200006658 | -1.4 |
| 10194 | TSHZ1 | teashirt zinc finger homeobox 1 | 223282_at | -1.6 | H200008758 | -1.1 |
| 10395 | DLC1 | deleted in liver cancer 1 | 220512_at | -1.9 | H200013095 | -1.6 |
| 10492 | SYNCRIP | synaptotagmin binding, cytoplasmic RNA interacting protein | 1555427_s_at | -2.2 | H200020157 | -1.8 |
| 10605 | PAIP1 | poly(A) binding protein interacting protein 1 | 208051_s_at | -2.8 | H200011655 | -1.3 |
| 11052 | CPSF6 | cleavage and polyadenylation specific factor 6, 68kDa | 202470_s_at | -1.8 | H200005547 | -1.6 |
| 22822 | PHLDA1 | pleckstrin homology-like domain, family A, member 1 | 217997_at | -3.4 | H200006885 | -1.4 |
| 22929 | SEPHS1 | selenophosphate synthetase 1 | 208939_at | -1.5 | H200012483 | -1.8 |
| 22943 | DKK1 | dickkopf homolog 1 (Xenopus laevis) | 204602_at | -2.1 | H200004487 | -1.3 |
| 22998 | LIMCH1 | LIM and calponin homology domains 1 | 212327_at | -3.9 | H200014950 | -1.7 |
| 23012 | STK38L | serine/threonine kinase 38 like | 212565_at | -4.0 | H200014292 | -1.5 |
| 23035 | PHLPP2 | PH domain and leucine rich repeat protein phosphatase 2 | 213407_at | -2.3 | H200008134 | -1.2 |
| 23047 | PDS5B | PDS5, regulator of cohesion maintenance, homolog B (S. cerevisiae) | 204742_s_at | -1.6 | H200007767 | -2.2 |
| 23174 | ZCCHC14 | zinc finger, CCHC domain containing 14 | 212655_at | -1.5 | H200010191 | -1.2 |
| 23250 | ATP11A | ATPase, class VI, type 11A | 230875_s_at | -1.7 | H200003896 | -1.2 |
| 23531 | MMD | monocyte to macrophage differentiation-associated | 203414_at | -1.4 | H200006727 | -2.2 |
| 23545 | ATP6V0A2 | ATPase, H+ transporting, lysosomal V0 subunit a2 | 229572_at | -4.3 | H200002163 | -1.3 |
| 25801 | GCA | grancalcin, EF-hand calcium binding protein | 203765_at | -1.5 | H200006685 | -1.2 |
| 26135 | SERBP1 | SERPINE1 mRNA binding protein 1 | 217725_x_at | -1.6 | H200007642 | -1.2 |
| 26575 | RGS17 | regulator of G-protein signaling 17 | 220334_at | -1.6 | H200016654 | -1.5 |
| 27161 | EIF2C2 | eukaryotic translation initiation factor 2C, 2 | 213310_at | -3.0 | H200014538 | -1.7 |
| 27295 | PDLIM3 | PDZ and LIM domain 3 | 238592_at | -1.8 | H200013168 | -1.3 |
| 27436 | EML4 | echinoderm microtubule associated protein like 4 | 220386_s_at | -2.3 | H200019486 | -1.6 |
| 29843 | SENP1 | SUMO1/sentrin specific peptidase 1 | 226619_at | -1.8 | H200005613 | -1.5 |
| 51167 | CYB5R4 | cytochrome b5 reductase 4 | 219079_at | -1.7 | H200001099 | -1.3 |
| 51280 | GOLM1 | golgi membrane protein 1 | 217771_at | -1.8 | H200008623 | -1.3 |
| 51444 | RNF138 | ring finger protein 138 | 239143_x_at | -1.7 | H200008430 | -1.6 |
| 51582 | AZIN1 | antizyme inhibitor 1 | 212461_at | -1.6 | H200015251 | -1.3 |
| 54149 | C21ORF91 | chromosome 21 open reading frame 91 | 226109_at | -2.0 | H200004933 | -1.8 |
| 54619 | CCNJ | cyclin J | 219470_x_at | -2.3 | H200017586 | -1.5 |
| 54855 | FAM46C | family with sequence similarity 46, member C | 226811_at | -1.7 | H200014087 | -1.5 |
| 54898 | ELOVL2 | elongation of very long chain fatty acids (FEN1/Elo2, SUR4/Elo3, yeast)-like 2 | 213712_at | -3.4 | H200015837 | -1.8 |
| 55007 | FAM118A | family with sequence similarity 118, member A | 226475_at | -1.6 | H200016550 | -1.4 |
| 55629 | PNRC2 | proline-rich nuclear receptor coactivator 2 | 217779_s_at | -1.5 | H200001509 | -1.6 |
| 55691 | FRMD4A | FERM domain containing 4A | 225163_at | -1.8 | H200014204 | -1.6 |
| 55692 | LUC7L | LUC7-like (S. cerevisiae) | 220143_x_at | -1.7 | H200002612 | -1.2 |
| 55704 | CCDC88A | coiled-coil domain containing 88A | 239233_at | -1.7 | H200002938 | -1.5 |
| 55740 | ENAH | enabled homolog (Drosophila) | 228310_at | -3.2 | H200002416 | -1.6 |
| 56261 | GPCPD1 | hypothetical protein KIAA1434 | 224835_at | -1.5 | H200008005 | -1.7 |
| 57154 | SMURF1 | SMAD specific E3 ubiquitin protein ligase 1 | 212666_at | -1.5 | H200018341 | -1.3 |
| 57157 | PHTF2 | putative homeodomain transcription factor 2 | 1554822_at | -3.4 | H200012762 | -1.4 |
| 57494 | RIMKLB | ribosomal modification protein rimK-like family member B | 225978_at | -2.0 | H200015566 | -1.2 |
| 57568 | SIPA1L2 | signal-induced proliferation-associated 1 like 2 | 225056_at | -4.3 | H200002761 | -1.6 |
| 60436 | TGIF2 | TGFB-induced factor homeobox 2 | 216262_s_at | -1.8 | H200010661 | -1.2 |
| 64388 | GREM2 | gremlin 2, cysteine knot superfamily, homolog (Xenopus laevis) | 240509_s_at | -10.9 | H200015021 | -2.2 |
| 65108 | MARCKSL1 | MARCKS-like 1 | 200644_at | -2.2 | H200005990 | -1.3 |
| 79677 | SMC6 | structural maintenance of chromosomes 6 | 236535_at | -1.9 | H200004266 | -1.7 |
| 79805 | VASH2 | vasohibin 2 | 219740_at | -2.0 | H200012574 | -1.9 |
| 80055 | PGAP1 | post-GPI attachment to proteins 1 | 213469_at | -1.7 | H200003487 | -1.4 |
| 80143 | SIKE1 | suppressor of IKK epsilon | 204666_s_at | -2.1 | H200009221 | -1.3 |
| 80155 | NAA15 | NMDA receptor regulated 1 | 226998_at | -2.4 | H200012532 | -1.4 |
| 80218 | NAA50 | N-acetyltransferase 13 (GCN5-related) | 222393_s_at | -1.5 | H200009413 | -1.2 |
| 80335 | WDR82 | WD repeat domain 82 | 201934_at | -1.6 | H200014605 | -1.3 |
| 81573 | ANKRD13C | ankyrin repeat domain 13C | 1556361_s_at | -2.3 | H200003173 | -1.3 |
| 83699 | SH3BGRL2 | SH3 domain binding glutamic acid-rich protein like 2 | 225354_s_at | -2.4 | H200001743 | -1.3 |
| 84168 | ANTXR1 | anthrax toxin receptor 1 | 224694_at | -2.0 | H200001715 | -1.9 |
| 84668 | FAM126A | family with sequence similarity 126, member A | 227239_at | -1.6 | H200003335 | -1.5 |
| 84823 | LMNB2 | lamin B2 | 216952_s_at | -1.5 | H200019661 | -2.2 |
| 84883 | AIFM2 | apoptosis-inducing factor, mitochondrion-associated, 2 | 228445_at | -1.5 | H200012032 | -1.4 |
| 84890 | ADO | 2-aminoethanethiol (cysteamine) dioxygenase | 212502_at | -1.9 | H200010994 | -1.2 |
| 85352 | KIAA1644 | KIAA1644 | 52837_at | -1.9 | H200001311 | -1.4 |
| 90007 | MIDN | midnolin | 225954_s_at | -1.7 | H200009932 | -1.2 |
| 90102 | PHLDB2 | pleckstrin homology-like domain, family B, member 2 | 225688_s_at | -1.6 | H200001424 | -2.5 |
| 93664 | CADPS2 | Ca++-dependent secretion activator 2 | 219572_at | -2.7 | H200011539 | -1.2 |
| 116150 | NUS1 | nuclear undecaprenyl pyrophosphate synthase 1 pseudogene | 225070_at | -1.7 | H200009444 | -1.6 |
| 147991 | DPY19L3 | dpy-19-like 3 (C. elegans) | 225633_at | -1.5 | H200003958 | -1.4 |
| 151050 | C2ORF67 | chromosome 2 open reading frame 67 | 231252_at | -2.1 | H200018855 | -1.2 |
| 154810 | AMOTL1 | angiomotin like 1 | 225459_at | -1.6 | H200011128 | -1.8 |
| 166968 | MIER3 | mesoderm induction early response 1, family member 3 | 231975_s_at | -1.6 | H200008856 | -1.4 |
| 169200 | TMEM64 | transmembrane protein 64 | 225972_at | -1.6 | H200010285 | -1.2 |
| 285590 | SH3PXD2B | SH3 and PX domains 2B | 231823_s_at | -1.8 | H200010133 | -1.3 |
| 338645 | LUZP2 | leucine zipper protein 2 | 215323_at | -2.5 | H200014684 | -2.1 |
| 339287 | MSL1 | male-specific lethal 1 homolog (Drosophila) | 212708_at | -1.7 | H200020500 | -1.3 |

^1^ Fold change calculated by SAM analysis of microarrays from young and senescent IMR90 fibroblasts.

^2^ Fold change calculated by limma analysis of microarrays from young and senescent MRC5 fibroblasts.
