# Supplementary material for: Deep Sequencing Reveals Novel MicroRNAs and Regulation of MicroRNA Expression during Cell Senescence
Source: PLoS One. 2011 May 26;6(5):e20509. doi: 10.1371/journal.pone.0020509 (PMC3102725; doi:10.1371/journal.pone.0020509)
Supplement: Table S6 — Potential target genes upregulated by senescence-induced miRNA underexpression in both types of fibroblasts, IMR90 and MRC5. (DOC) [file pone.0020509.s007.doc]

**Table S6**. Potential target genes upregulated by senescence-induced miRNA underexpression in both types of fibroblasts, IMR90 and MRC5.

|  | | | **IMR90 fibroblast (Affymetrix)** | | **MRC5 fibroblast (two-color microarrays)** | |
| --- | --- | --- | --- | --- | --- | --- |
| **Entrez gene id** | **Gene symbol** | **Gene name** | **Probe id** | **FC^1^** | **Probe id** | **FC^2^** |
| 205 | AK4 | adenylate kinase 3-like 2; adenylate kinase 3-like 1 | 225342_at | 8.0 | H200001948 | 2.1 |
| 586 | BCAT1 | branched chain aminotransferase 1, cytosolic | 214452_at | 2.3 | H200018892 | 1.2 |
| 858 | CAV2 | caveolin 2 | 203323_at | 1.6 | H200013312 | 1.5 |
| 961 | CD47 | CD47 molecule | 211075_s_at | 1.8 | H200006967 | 1.4 |
| 1601 | DAB2 | disabled homolog 2, mitogen-responsive phosphoprotein (Drosophila) | 232898_at | 1.6 | H200006868 | 1.4 |
| 1605 | DAG1 | dystroglycan 1 (dystrophin-associated glycoprotein 1) | 205417_s_at | 1.6 | H200006252 | 1.3 |
| 2035 | EPB41 | erythrocyte membrane protein band 4.1 (elliptocytosis 1, RH-linked) | 225051_at | 1.8 | H200004395 | 1.4 |
| 3339 | HSPG2 | heparan sulfate proteoglycan 2 | 201655_s_at | 1.7 | H200015112 | 1.3 |
| 3655 | ITGA6 | integrin, alpha 6 | 215177_s_at | 2.6 | H200015396 | 2.5 |
| 4124 | MAN2A1 | mannosidase, alpha, class 2A, member 1 | 205105_at | 2.0 | H200004199 | 1.3 |
| 4644 | MYO5A | myosin VA (heavy chain 12, myoxin) | 204527_at | 2.1 | H200007910 | 1.6 |
| 4779 | NFE2L1 | nuclear factor (erythroid-derived 2)-like 1 | 200758_s_at | 1.9 | H200007037 | 1.5 |
| 5066 | PAM | peptidylglycine alpha-amidating monooxygenase | 202336_s_at | 2.0 | H200007074 | 1.6 |
| 5420 | PODXL | podocalyxin-like | 201578_at | 1.7 | H200002575 | 2.0 |
| 5865 | RAB3B | RAB3B, member RAS oncogene family | 239202_at | 2.4 | H200002936 | 1.5 |
| 6781 | STC1 | stanniocalcin 1 | 204597_x_at | 2.1 | H200003541 | 2.7 |
| 6867 | TACC1 | transforming, acidic coiled-coil containing protein 1 | 217433_at | 1.9 | H200008118 | 1.5 |
| 7328 | UBE2H | ubiquitin-conjugating enzyme E2H (UBC8 homolog, yeast) | 221962_s_at | 1.9 | H200003845 | 1.5 |
| 7832 | BTG2 | BTG family, member 2 | 201236_s_at | 1.8 | H200006111 | 1.7 |
| 8091 | HMGA2 | high mobility group AT-hook 2 | 1558682_at | 1.8 | H200000670 | 3.0 |
| 9076 | CLDN1 | claudin 1 | 222549_at | 2.1 | H200001413 | 3.8 |
| 9411 | ARHGAP29 | Rho GTPase activating protein 29 | 203910_at | 2.6 | H200005729 | 1.8 |
| 9648 | GCC2 | GRIP and coiled-coil domain containing 2 | 202832_at | 1.6 | H200017335 | 1.2 |
| 10140 | TOB1 | transducer of ERBB2, 1 | 202704_at | 1.8 | H200008309 | 1.7 |
| 10370 | CITED2 | Cbp/p300-interacting transactivator, with Glu/Asp-rich carboxy-terminal domain, 2 | 209357_at | 2.2 | H200006882 | 1.2 |
| 22906 | TRAK1 | trafficking protein, kinesin binding 1 | 202079_s_at | 1.6 | H200020132 | 1.9 |
| 23057 | NMNAT2 | nicotinamide nucleotide adenylyltransferase 2 | 1552712_a_at | 2.3 | H200007305 | 1.3 |
| 25827 | FBXL2 | F-box and leucine-rich repeat protein 2 | 214436_at | 1.8 | H200001149 | 2.2 |
| 26100 | WIPI2 | WD repeat domain, phosphoinositide interacting 2 | 204710_s_at | 1.9 | H200015347 | 1.4 |
| 55531 | ELMOD1 | ELMO/CED-12 domain containing 1 | 231930_at | 1.8 | H200004878 | 1.5 |
| 56204 | KIAA1370 | KIAA1370 | 225327_at | 1.9 | H200003947 | 2.0 |
| 64786 | TBC1D15 | TBC1 domain family, member 15 | 218268_at | 1.9 | H200004904 | 1.4 |
| 80031 | SEMA6D | sema domain, transmembrane domain (TM), and cytoplasmic domain, (semaphorin) 6D | 233882_s_at | 5.6 | H200014470 | 1.5 |
| 83464 | APH1B | anterior pharynx defective 1 homolog B (C. elegans) | 221036_s_at | 1.7 | H200004606 | 1.6 |
| 94241 | TP53INP1 | tumor protein p53 inducible nuclear protein 1 | 225912_at | 1.8 | H200006117 | 1.7 |
| 160518 | DENND5B | DENN/MADD domain containing 5B | 228551_at | 1.6 | H200004055 | 1.3 |

^1^ Fold change calculated by SAM analysis of microarrays from young and senescent IMR90 fibroblasts.

^2^ Fold change calculated by limma analysis of microarrays from young and senescent MRC5 fibroblasts.
